# The role of extracellular vesicles in periodontitis: pathogenesis, diagnosis, and therapy

**DOI:** 10.3389/fimmu.2023.1151322

**Published:** 2023-04-11

**Authors:** Rong Cai, Lu Wang, Wei Zhang, Bing Liu, Yiqi Wu, Jianliang Pang, Chufan Ma

**Affiliations:** ^1^ Department of Stomatology, Air Force Medical Center, The Fourth Military Medical University, Beijing, China; ^2^ Department of Critical Care Medicine, The First Medical Center, Chinese PLA General Hospital, Beijing, China

**Keywords:** periodontitis, extracellular vesicles, stem cell, pathogenesis, diagnosis

## Abstract

Periodontitis is a prevalent disease and one of the leading causes of tooth loss. Biofilms are initiating factor of periodontitis, which can destroy periodontal tissue by producing virulence factors. The overactivated host immune response is the primary cause of periodontitis. The clinical examination of periodontal tissues and the patient’s medical history are the mainstays of periodontitis diagnosis. However, there is a lack of molecular biomarkers that can be used to identify and predict periodontitis activity precisely. Non-surgical and surgical treatments are currently available for periodontitis, although both have drawbacks. In clinical practice, achieving the ideal therapeutic effect remains a challenge. Studies have revealed that bacteria produce extracellular vesicles (EVs) to export virulence proteins to host cells. Meanwhile, periodontal tissue cells and immune cells produce EVs that have pro- or anti-inflammatory effects. Accordingly, EVs play a critical role in the pathogenesis of periodontitis. Recent studies have also presented that the content and composition of EVs in saliva and gingival crevicular fluid (GCF) can serve as possible periodontitis diagnostic indicators. In addition, studies have indicated that stem cell EVs may encourage periodontal regeneration. In this article, we mainly review the role of EVs in the pathogenesis of periodontitis and discuss their diagnostic and therapeutic potential.

## 1 Introduction

Periodontal disease is the sixth most prevalent disease in the world (1, 2). According to a 2017 report, periodontitis affects 796 million people worldwide (3), places a substantial financial and health burden on those affected, and drastically lowers their quality of life (4, 5).

In recent years, risk factors of periodontitis have been grouped into three main categories: biofilms, host, and environment (6). When local biofilms and the mild host immune are in balance, the immune surveillance and appropriate immune response predominate (7). When exposed to persistent microbial challenges or when the pathogenicity of the local microbiome increases, the balance between biofilms and the host is lost, and the host’s immune reactivity is excessive. This results in a highly inflammatory state with immune cell infiltration, pro-inflammatory and inflammatory cytokines up-regulation, excessive osteoclasts activation, ligament fiber degradation, granulation tissue formation, and final periodontal destruction (8–16).

Clinical features of periodontitis include red, swollen, and receding gums, bleeding on periodontal probing, a deeper pocket, the destruction of periodontal tissue, tooth displacement, and eventually tooth loss (17, 18). Unfortunately, due to the low sensitivity and low positive predictive value of these tests, the parameters can only evaluate historical data on periodontal tissue loss and cannot forecast future disease activity (19–21). Furthermore, these parameters vary among dentists, which impacts the accuracy of diagnosing periodontitis (22). To prevent and diagnose periodontitis early and effectively refer patients to specialized therapy, it is crucial to investigate more repeatable, sensitive, and specific methods of periodontal diagnosis that provide current and future disease information (23, 24).

Periodontitis treatment comprises non-surgical treatment, surgical treatment, and adjuvant medicine treatment (25–27). The treatment objectives are to control inflammation, halt disease progression, and help patients reconstruct a healthy and functional dentition (28). Surgical intervention is required when it is necessary to rebuild a bone defect to establish a good bone structure or when regeneration is needed to restore lost periodontal structures (29). Periodontal regeneration is a complex process due to the unique anatomical structure and composition of periodontal tissue, including periodontal ligament, cementum, and alveolar bone (30). Osteogenesis, inflammation control, and angiogenesis play important roles in periodontal regeneration (31, 32). At present, guided tissue regeneration techniques, including the transplantation of soft and hard tissues, the use of growth factors and host regulatory factors, and the use of biomaterials, are the mainstays of periodontal regeneration (33–35). Nevertheless, there are just a few materials with high potential for periodontal regeneration, and present technologies have limitations in attaining periodontal regeneration. Thus, looking for more durable and potent therapies and materials is critical to improving periodontal regeneration (36–38).

EVs are a group of bilayered lipid membrane-structured vesicles secreted by multiple kinds of cells. They carry a variety of substances from the parental cells, like DNA, RNA, lipids, and proteins (23, 39). EVs play a role in various pathological and physiological processes, including immunological regulation, inflammatory response, and tissue healing and regeneration. EVs have been discovered valuable to study physiological processes, pathologies, as well as regeneration (40).

We outlined the function of EVs in the pathogenesis and diagnosis of periodontitis and discussed methods used to isolate and characterize salivary and GCF EVs in this paper. We also reviewed recent research on stem cell-derived EVs in periodontitis therapy and addressed the flaws and future directions.

## 2 Extracellular vesicles

### 2.1 Definition of EVs

EVs can be secreted by humans, plants, animals, and microbial origins (40). Only a few nonmammalian sources have been explored in preclinical or clinical settings, and most studies and reviews have concentrated on EVs derived from mammalian cells and body fluids (40). EVs can be classified as endosome-derived exosomes (Exo), plasma membrane-derived microvesicles (MVs), and apoptotic bodies (ApoEVs) based on their secretion processes and characteristics (41, 42). Exosomes (30-100 nm) germinate inward from the endosome membrane, forming multivesicular bodies (MVBs) in the cytoplasm. Some MVBs are degraded by lysosomes, while others fuse with the cell membrane and are discharged into the extracellular environment by exocytosis. MVs (50 nm-2μm) and ApoEVs (50 nm-5 μm) were derived from outward budding. Exosomes and MVs are secreted during normal cellular processes, whereas ApoEVs are only produced during programmed cell death (43–46). In 2018, the International Extracellular Vesicle Society recommended researchers characterize EVs by size: “small EVs” (sEVs < 200 nm) and “medium and/or large EVs” (m/lEVs > 200 nm) unless specific EVs markers are available (47).

EVs can participate in physiological and pathological processes by directly binding to receptors on recipient cells, fusing with the plasma membrane of recipient cells and the membrane of the endosome following endocytosis (48, 49). Various endocytosis-related pathways, including clathrin-dependent endocytosis and clathrin-independent pathways such as caveolin-mediated uptake, macropinocytosis, phagocytosis, and lipid raft-mediated internalization, are thought to be the primary mechanisms of EVs uptake by recipient cells (48, 50).

### 2.2 Functions of EVs

In physiological and pathological processes, including cancer treatment, early diagnosis, tissue regeneration, and medication, the released EVs can remove metabolic proteins during cell maturation and regulate cell-to-cell communication (39, 51–54).

The RNA composition of EVs varies with pathological situations, and as a result, they have become a source of biomarkers for diagnosing human diseases (55). Proteins, genetic material, and lipids in EVs extracted from oral biofluids (saliva and GCF) have recently emerged as potential sources of biomarkers for periodontal diseases (23).

On the other hand, EVs are used in disease therapy in that the biological properties of EVs can modulate the phenotype and behavior of recipient cells (56, 57). Due to their innate ability to promote tissue regeneration, mesenchymal stem cells (MSCs) have been employed as a source of regenerative EVs. Besides, MSCs-EVs have the following advantages (1): MSCs-EVs have the innate capacity to cross physiological barriers, such as the blood-brain barrier, due to their nanoscale size (58, 2). The risk of immune rejection and tumorigenicity induced by cell transplantation can be decreased with MSCs-EVs therapy (59, 3). MSCs-EVs are highly stable and biocompatible, and recipient cells may quickly absorb them (60, 4). MSCs-EVs are more convenient to store and transport since they can be kept stable at low temperatures (61, 5). Appropriate modification can enhance the targeting and repair abilities of MSCs-EVs (50, 62, 6). Studies have also demonstrated that MSCs-EVs have no adverse effects in toxicology tests (63, 7). MSCs-EVs are comparable to MSCs in their capacity to repair injured tissues, resist inflammation, inhibit cell apoptosis, and regulate immune responses (64–67). *In vitro* and animal studies have shown the potential of MSCs-derived EVs for treating periodontitis (68, 69). MSCs-EVs have demonstrated potential in the prevention and treatment of periodontal disease as well as periodontal regeneration due to their ability to regulate inflammation and promote osteogenesis (70, 71).

### 2.3 Extraction and characterization of EVs

Five basic extraction techniques are frequently employed based on the physical (density, size, and solubility) and biological (surface antigen) properties of EVs: precipitation, membrane affinity, size-exclusion chromatography, iodixanol gradient, and phosphatidylserine affinity (72). Additional methods have been developed to improve the specificity of EVs, including tangential flow filtration (73), field-flow fractionation (74), asymmetric flow field-flow fractionation (75), field-free viscoelastic flow (76), alternating current electrophoretic (77), acoustics (78), ion exchange chromatography (75), microfiltration (79), fluorescence-activated sorting (80), etc.

According to the latest MISEV 2018 guidelines for EVs characterization, scientists should include at least three distinct characteristics, such as EVs particle quantity, morphology, and protein markers (47). Dynamic light scattering (81) and nanoparticle tracking analysis (82) are frequently applied to estimate the quantity and size of EVs particles. Transmission electron microscopy (83), scanning electron microscopy (84), and atomic force microscopy (85) can be used to examine the morphology of EVs. Bicinchoninic acid assay (86), fluorimetric assays (87), or the global protein stain on SDS-PAGE (88) were used for EVs protein quantification. Western blot (89), enzyme-linked immunosorbent assay (90), bead-based flow cytometry (91), aptamer- and carbon nanotube-based colorimetric assays (92), and surface plasmon resonance (93) were employed to detect protein markers. Generally, at least one protein from each of the following groups must be assessed (47): (1) Transmembrane or GPI-anchored proteins connected to the plasma membrane or endosomes (Tetraspanins, integrins, etc.). (2) Cytosolic proteins (membrane binding proteins, etc.). (3) Major non-EVs co-isolated structural constituents (lipoproteins, Apolipoproteins, ribosomal proteins, etc.). When claiming specific analysis of sEVs, analysis of transmembrane, lipid-bound and soluble proteins associated with other intracellular compartments (histones, cytochrome C, etc.) is required. Secreted proteins recovered with EVs (cytokines and growth factors, adhesion, extracellular matrix proteins, etc.) are needed to document the functional activities of sEVs. To further indicate particle per volume and particle size distribution, the guidelines also suggested the addition of EVs purity metrics, such as protein/particle ratio, protein/lipid ratio, or RNA/particle ratio.

## 3 EVs and the pathogenesis of periodontitis

### 3.1 Direct pathogenic role of outer-membrane vesicles in periodontitis

The Gram-negative bacteria that are closely related to the progression of periodontitis, including Porphyromonas *gingivalis (P. gingivalis), Treponema denticola (T. denticola), Tannerella forsythia (T. forsythia), Actinomyces reticulata (A. reticulata), Fusobacterium nucleatum (F. nucleatum) and Prevotella intermedia (P. intermedia)* have been isolated from the periodontal pocket (94–96). *P. gingivalis* is the primary pathogen responsible for chronic periodontitis (97, 98). It forms the “red complex” along with *T. forsythia* and *T. denticola*, which are accessory pathogens with complementary or supplementary functions (99, 100).

Gram-negative bacteria can selectively export toxins and other virulence factors to host cells through vesicles named OMVs. In light of our current knowledge, OMVs are double-layered spherical membrane-like structures with a diameter ranging from 20 to 250 nm. OMVs contain bacterial parts and products such as fimbriae, lipopolysaccharides (LPS), toxins, outer membrane proteins, peptidoglycans, and bacteria’s DNA and RNA (101–107). Yet it is unclear how these elements are packed into OMVs, and how the cargos are selected (101). OMVs can directedly fuse with target cells or be internalized by lipid rafts, micropinocytosis, and clathrin-dependent endocytosis (108–110).

After entering host cells, OMVs can exhibit a variety of virulences (111), and host-derived proteases have little effect on them (112). While requiring much energy, OMVs are crucial for maintaining bacterial virulence, colony formation, material transfer inside bacteria, immune escape, and host cell immune regulation (101, 103, 113–116).

The gingival epithelium is a physical barrier against invasion by biofilms and other nonautologous substances and is the first line of defense in the oral cavity (117, 118). There have been reports of *P. gingivalis* OMVs invading oral epithelial cells (119). By the endocytic pathway, *P. gingivalis* OMVs can efficiently infiltrate human epithelial cells and interfere with their function by destroying signaling molecules necessary for cell migration, such as transferrin receptor, paxillin, and focal adhesion kinase (120, 121). *T. denticola* can disrupt the function of the epithelial barrier and penetrate the epithelial layers by degrading tight junctional proteins like ZO-1 (122). According to Bartruff (123) et al., OMVs derived from *P. gingivalis* significantly inhibited the proliferation of cultured gingival fibroblasts and human umbilical vein endothelial cells (hUVECs), as well as hUVECs’ ability to form capillaries, which restrained periodontal tissue healing.

OMVs could be oral microbial communication between *P. gingivalis* and other oral bacteria (119). Kamaguchi (124) et al. demonstrated that *P. gingivalis* OMVs significantly promoted oral bacteria coaggregation. Grenier (125) noticed that *P. gingivalis* OMVs could mediate the coaggregation between *T. denticola* and *L. saburreum*. *P. gingivalis* and *T. denticola* co-inoculation synergistically triggered host immune responses and alveolar bone loss in a murine experimental periodontitis model (126). According to Inagaki (127) et al., *P. gingivalis* OMVs play an important role in virulence by enhancing *T. forsythia’s* adherence and penetration of epithelial cells. *P. gingivalis* OMVs have explicitly been enriched for the heme-binding lipoproteins HmuY and IhtB, which can provide micronutrients to several other subgingival biofilms, resulting in community benefits that encourage biofilm proliferation (128). In addition, *P. gingivalis* OMVs can suppress and disperse rival biofilms in a gingipains-dependent manner to create a favorable environment for *P. gingivalis* (119).

**Figure 1:**
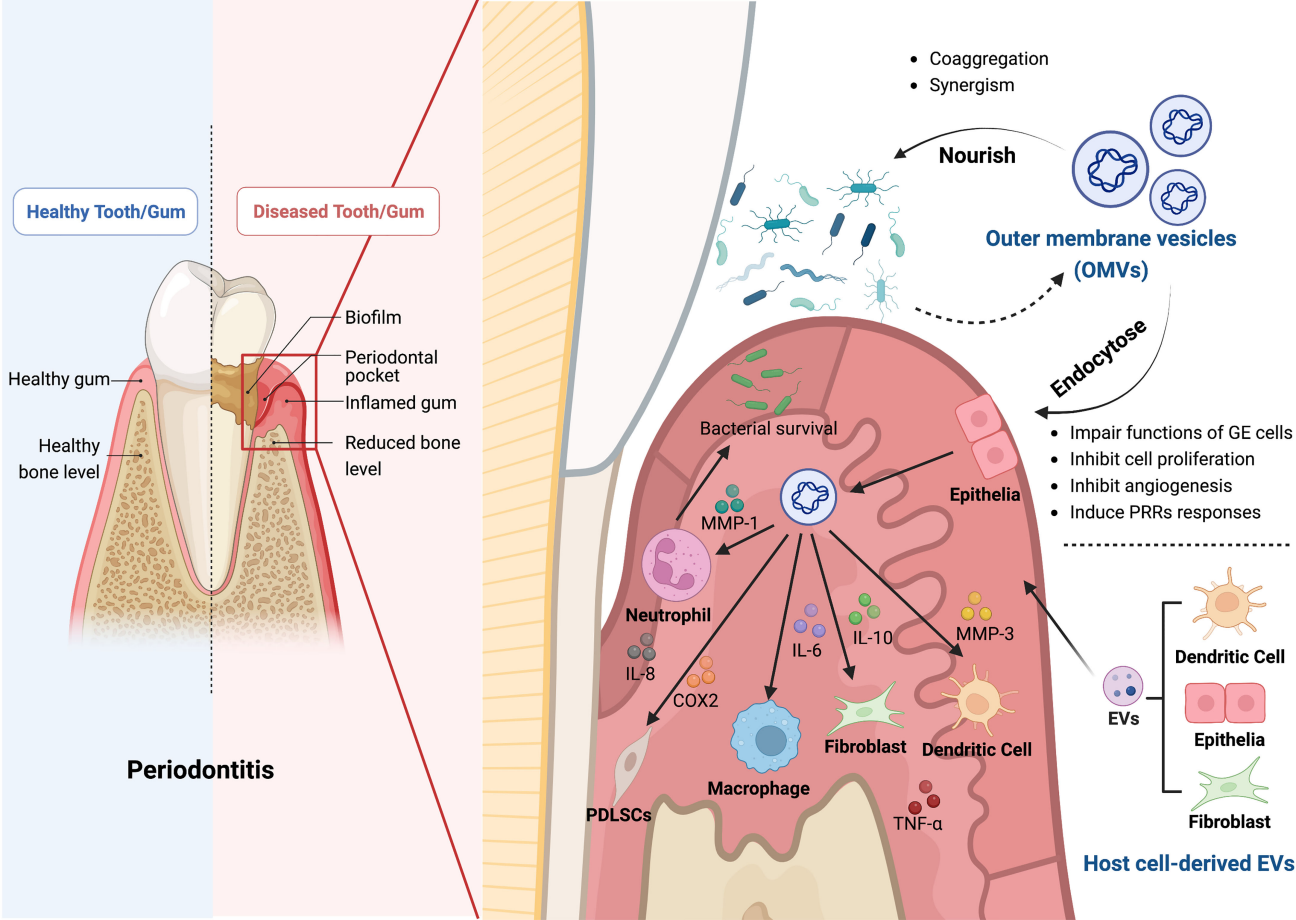
The roles of bacterial outer membrane vesicles (OMVs) and host cell-derived EVs in the pathogenesis of periodontitis. OMVs produced by Gram-negative bacteria contain bacterial components and bacterial products, such as outer membrane proteins, peptidoglycan, and lipopolysaccharide, which play a crucial role in the pathogenesis of periodontitis. Firstly, OMVs can enter the gingival epithelial cells through endocytosis and thus impair the function of gingival epithelial cells, inhibiting epithelial cell proliferation, slowing down angiogenesis, and inducing the response to PRRs. Secondly, OMVs can also inhibit bacterial clearance by immune cells by affecting a variety of cellular functions through different inflammatory mediators, including neutrophils, macrophages, fibroblasts, periodontal stem cells, and dendritic cells, which impede the host immune response. OMVs can cause aggregation of bacteria and act synergistically with them to induce the onset and progression of periodontitis. In addition, the EVs secreted by the host cells, such as dendritic cells, fibroblasts, and epithelial cells, can also cause alveolar bone loss, periodontitis and tissue damage. The Graph was created with BioRender.com. PRRs, pattern recognition receptors; MMP, matrix metallopeptidase; IL, interleukin; COX2, cyclooxygenase 2; TNF-α, tumor necrosis factor-α.

In brief, these findings indicate that OMVs, which can mediate the interaction between biofilms and host cells and hasten the destruction of periodontal tissue, are substantially responsible for the pathogenicity of biofilms (**Figure 1**). Nevertheless, the precise mechanisms by which OMVs alter the nature of biofilms remain unclear. Further research is required to determine the specific function and associated mechanisms of OMVs in periodontitis.

### 3.2 Pathogenic role of OMVs in periodontitis by affecting immunity and inflammation

Pattern recognition receptors (PRRs), expressed by host immune cells, are essential molecules that trigger local immune responses. OMVs from periodontal pathogens can induce PRRs reactions (112). OMVs can activate PRRs in gingival epithelial cells, causing the secretion of pro-inflammatory and anti-inflammatory cytokines and activating neutrophils, T and B lymphocytes, and osteoclasts. These reactions promote connective tissue destruction and alveolar bone resorption (129). Choi (130) et al. derived that OMVs secreted by major periodontal pathogens transferred microRNA (miRNA) to immune cells to suppress target genes related to immune response, thereby evading the host adaptive immune responses. The potent but flexible immunostimulatory effects of *P. gingivalis* OMVs may help manipulate and dysregulate host immune responses to initiate disease, and the pro-inflammatory effects of other bacteria may contribute to the disease progression (100). *P. gingivalis* OMVs can selectively promote tumor necrosis factor (TNF) tolerance in a Toll-Like Receptor 4 and mTOR-Dependent manner, leading to local immune evasion (131). *P. gingivalis* OMVs can selectively entrap and activate human neutrophils to initiate degranulation without being destroyed by neutrophils, and they can also breakdown components of secretory granules with antibacterial activity, especially LL-37 and myeloperoxidase (MPO), to ensure bacterial survival (132).

The levels of inflammatory mediators, particularly interleukin (IL)-1, TNF, prostaglandin E2, and cyclooxygenase-2 (COX-2), are correlated with the severity of inflammatory response and periodontal disease (133, 134). Kou (135) et al. discovered that co-culturing immortalized human gingival epithelial cells with *P. gingivalis* OMVs elevated the production of inflammatory factors as COX-2, IL-6, IL-8, matrix metalloproteinase (MMP)-1, and MMP-3. Another study demonstrated that *P. gingivalis* OMVs enhanced the expression of IL-6 and IL-8 in human gingival epithelial cells *via* activating the signaling pathways Erk1/2, JNK, MAPK, STING, and NF-κB (136). Fleetwood (137) et al. confirmed that *P. gingivalis* OMVs could penetrate the gingival tissue and stimulate macrophages to produce large amounts of TNF-α, IL-12, IL-6, IL-10, IFN-β, and NO, resulting in tissue inflammation and damage. OMVs from *P. gingivalis* and *T. forsythia* induced the expression of pro-inflammatory cytokines like IL-1β, IL-6, IL-23, and IL-12p70 in bone marrow-derived dendritic cells (DCs) (138). Human monocyte cell line U937 and periodontal ligament fibroblasts were activated by *T. forsythia* OMVs in a concentration-dependent manner to produce pro-inflammatory mediators, and the inflammatory response was noticeably greater than that induced by whole *T. forsythia* cells (139).

OMVs should be considered as part of a larger picture because they not only contribute to the local problem of periodontitis (40). As EVs communication is not confined to species, OMVs from periodontal pathogens are also involved in human systemic diseases (104), for instance, Alzheimer’s disease (140), neuroinflammation and neurodegeneration (141), cardiovascular disease (142), and diabetes mellitus (98). Investigating interkingdom communication of EVs from different origins may help discover new pathologic mechanisms and innovative therapies.

OMVs derived from probiotic strains contain immunomodulatory molecules that decrease pro-inflammatory cytokines and strengthen epithelial barriers (143, 144). According to reports, OMVs are protease resistant, can withstand long-term storage, and their structural stability makes it easier for them to deliver contents into the host immune system. Moreover, OMVs are attractive vaccines against pathogenic bacteria due to their immunogenicity (145, 146). Specific antibodies against *P. gingivalis* can be produced in mice’s blood and saliva by intranasal inoculating OMVs (147, 148). Whereas, because they are still in the very early stages of development, periodontal vaccines face obstacles such as limited yield, unfavorable toxicity, and insufficient immunogenicity (149).

### 3.3 Host cell-derived EVs in the pathogenesis of periodontitis

Immune senescence plays a pivotal role in the pathophysiology of experimental periodontitis. *P. gingivalis* directly invades DCs to cause premature senescence and dramatically accelerates the senescence of normal bystander DCs by secreting inflammatory exosomes (150). Exosomes of the *P. gingivalis*-invaded DCs transmit senescence to normal bystander DCs and T cells, resulting in the loss of alveolar bone, according to recently published research (151). Another study demonstrated that biofilms could contribute to inflammation and periodontal destruction by promoting gingival fibroblasts to exhibit a tissue-destructive phenotype *via* increased secretion of epithelial EVs (152). Otherwise, LPS-treated periodontal ligament fibroblasts induce inflammation and inhibit the osteogenic activity of osteoblasts by releasing exosomes (153). Presumably, EVs secreted by host cells significantly impact periodontal disease.

In conclusion, OMVs from periodontal pathogens and host cell-derived EVs are critical in the pathogenesis of periodontitis. Nevertheless, there is still much to learn about the precise molecules and mechanisms by which EVs mediate innate and acquired immune response in periodontitis (6).

## 4 EVs and diagnosis of periodontitis

### 4.1 Diagnostic role of EVs in saliva and GCF

Saliva, a hypotonic solution composed of GCF, serum, salivary glands secretion, oral mucosal secretion, and microorganisms, is responsible for oral cleaning, antibacterial effect, and host’s resistance to oral infections (154, 155). Another important oral biofluid is GCF, a serum exudate of periodontal tissue, presented in the healthy gingival sulcus or the periodontal pocket. Saliva and GCF are rich in biomolecules from the host and microorganisms, such as inflammatory mediators, cytokines, tissue breakdown products, DNA, RNA, EVs, etc. (23, 154, 156–158). Therefore, saliva and GCF can be applied as promising non-invasive indicators for periodontitis (159).

Genetic analysis of saliva sEVs showed that innate immune response proteins were considerably enriched in patients with severe periodontitis, particularly the complement component 6 (C6) (160). One study proposed that the DNA methylation pattern of 5-methylcytosine (5mC) in saliva EVs was comparable in the groups with healthy gums, gingivitis, and periodontitis (161). According to another study, patients with periodontitis had considerably fewer CD9+ and CD81+ saliva exosomes than healthy controls (162). CD63+ exosomes in GCF were increased in patients with periodontitis compared with those without periodontitis (157). In addition to EVs surface markers, miRNAs were abundant in EVs, and the level of hsa-miR-199a-3p decreased with the development and progression of periodontitis (163). There was a decrease in miR-223-3p concentration in periodontitis (164). In contrast, periodontitis dramatically enhanced the expression of hsa-miR-140-5p, hsa-miR-146a-5p, hsa-miR-628-5p, and PD-L1 mRNA (165, 166). A *P. gingivalis* saliva diagnostic kit for the detection of *P. gingivalis* and *P. gingivalis* OMVs has been developed by researchers using monoclonal antibodies that identify the conserved *P. gingivalis* virulence factor RgpA-Kgp complex (167).

These findings demonstrate that EVs released by saliva and GCF can reveal alterations in the local microenvironment and may indicate periodontitis (157, 168, 169). Owing to that, saliva and GCF EVs may one day become simple and fast chair-side methods for diagnosing and evaluating periodontitis activity.

### 4.2 Collection of saliva and GCF and isolation of EVs

Patients should refrain from drinking and eating for at least one hour before sampling to avoid contamination of saliva and GCF with food and drink (23, 161, 163). Saliva can be collected either stimulated or unstimulated. Stimulated saliva is collected by chewing or gustatory stimulation (such as chewing paraffin or placing citric acid). In contrast, unstimulated saliva is collected by spitting or drooling without chewing or gustatory stimulation (170). Techniques used to collect saliva can affect its composition and the ultimate determination of particular biomarkers (171). As a result, saliva collection should closely resemble actual clinical conditions, and sample collection and processing should be consistent throughout.

Subgingival biofilms should be removed, and teeth should be blown dry to exclude saliva interference before GCF collection. After gently sampling with filter paper strips to prevent contamination from bleeding, GCF is eluted with PBS (172). Researchers should make it clear whether the GCF is from the healthy or the diseased site and correctly record the clinical parameters of each site, which is of great value in parsing the biochemical information (159, 168).

Although there is no optimal method for isolating saliva and GCF EVs, several researchers have compared different isolation protocols. In a comparison of saliva sEVs obtained by ultracentrifugation (UC) and ExoQuick-TC (TM) (EQ) precipitation, Zlotogorski - Hurvitz (173) et al. found that EQ generated a larger shape/aggregation pattern and a higher CD63/CD9/CD81+ sEVs subset than UC. Other investigators compared the particle production and particle/protein ratio of UC-sEVs and SEC-sEVs in saliva, showing that SEC-sEVs were superior in both categories (23).

In summary, mounting evidence points to the possibility that EVs generated from GCF and saliva may serve as vital diagnostic biomarkers for periodontitis owing to their cargo of proteins, RNA, and DNA (**Table 1**). But there is still a long way to go before EVs can be used for clinical diagnosis because the techniques for collecting EVs are currently only in-vitro or pre-clinical. The primary challenge is standardized techniques for isolating and characterizing EVs. More specific, sensitive, and practical biomarkers should be developed (174). An analysis with a large sample size is required to establish proper EVs-periodontitis diagnostic criteria matched with different ages, genders, etc.

**Table 1:** Application of exosomes in the diagnosis of periodontitis.

Author (Year)	Research type	Origin of EVs	Contents/markers	Groups	Sample size	Conclusions	Reference
Chaparro Padilla A, et al. (157)	cross-sectional case-control study	saliva, GCF	CD9, TSG101, Alix	Periodontitis(stages II, III, and IV)	41	hypersecretion, pro-inflammatory	(141)
				Gingival health or Gingivitis	45		
Huang X, et al. (160)	cross-sectional case-control study	saliva	CD9, CD81	Severe Periodontitis (SP)	11	protein expression difference, pro-inflammatory	(144)
				Periodontal health	11		
Han P, et al. (161)	cross-sectional case-control study	saliva	CD9, TSG101	Periodontitis	8	hypersecretion, pro-inflammatory	(145)
				Gingivitis	7		
				Periodontal health	7		
Tobon-Arroyave SI, et al. (162)	cross-sectional case-control study	saliva	CD9, CD81	Periodontitis	104	hyposecretion	(146)
				Periodontal health	45		
Nik Mohamed Kamal NNS, et al. (163)	cross-sectional case-control study	saliva, plasma	unspecified	Chronic Periodontitis(CP)	8	miRNA expression difference, expression downregulated	(147)
				Periodontal health	8		
Xia Y, et al. (164)	cross-sectional case-control study	saliva	miR-223-3p	Periodontitis(stages III and IV)	none	expression downregulated, anti-inflammatory	(148)
				Periodontal health	none		
Yu J, et al. (165)	prospective observational investigation	saliva	PD-L1	Periodontitis	61	expression upregulated, pro-inflammatory	(149)
				Periodontal health	30		
Han P, et al. (166)	cross-sectional case-control study	saliva	unspecified	Periodontitis(stages III and IV)	10	expression upregulated	(150)
				Gingivitis	9		
				Periodontal health	10		

## 5 MSCs-derived EVs and therapy of periodontitis

### 5.1 Role of MSCs-EVs in periodontitis treatment *via* anti-inflammatory and immune regulation

As an essential component of the innate immune system, macrophages mediate the onset and progression of periodontitis (175). Macrophages can differentiate into either a pro-inflammatory (M1) or an anti-inflammatory (M2) phenotype in reaction to local microenvironments, with each playing a unique role in a variety of physiological or pathological conditions (176, 177). Cytokines like TNF- and IL-6, which are produced by M1 macrophages, increase inflammation, activate osteoclasts, and result in the resorption of alveolar bone. In contrast, factors such as IL-10 and transforming growth factor (TGF) -β, produced by M2 macrophages, have anti-inflammatory and angiogenic effects and can activate osteoblasts (175, 176, 178, 179). Consequently, modulating the macrophage M1/M2 polarization ratio is an effective strategy for intervening in diseases.

In a rat calcaneus defect model, TNF-α-pretreated MSCs-EVs possess stronger immunomodulatory properties that can suppress M1 macrophages markers like IL-1β and iNOS and increase M2 macrophages markers like Arg1 and CD206, thereby promoting bone formation (180). MSCs-derived exosomes can improve the treatment of periodontitis by reestablishing the equilibrium of T helper 17/regulatory T cells (Th17/Treg) in inflamed periodontal tissues (181).

Bone mesenchymal stromal cells (BMSCs)-derived EVs regulate the inflammatory immune response and promote periodontal regeneration by inhibiting osteoclast activity, influencing macrophage polarization to M2, and regulating the production of TGF-β1 (68). Xu (182) et al. found that upon LPS stimulation, BMSCs-EVs converted macrophages from M1 to M2 phenotype *in vitro*, which decreased inflammation. In addition, ApoEVs derived from BMSCs can inhibit the polarization of macrophages into the M1 phenotype, reduce the COX-2 expression, down-regulate the TNF-α secretion, and inhibit adjacent osteoclasts, which serve as the foundation for the treatment of periodontitis (183). BMSCs-EVs have become promising therapeutic strategies for managing periodontitis (184).

The TNF-α-pretreated exosomes derived from gingiva mesenchymal stem cells (GMSCs) are notable for their ability to induce M2 polarization and prevent osteoclast formation (185). Wang (186) et al. observed that macrophages co-cultured with GMSCs exosomes in the inflammatory microenvironment showed significantly lower levels of M1 markers but somewhat raised levels of M2 features. In other words, GMSCs exosomes could trigger the transformation of M1 macrophages into M2 macrophages and lessen the pro-inflammatory substances that M1 macrophages release (186). Another study showed that GMSCs-sEVs significantly improved periodontal regeneration by inhibiting the release of pro-inflammatory cytokines from T cells and monocytes/macrophages, blocking T cells activation, and inducing the creation of Tregs (187).

Zheng (188) et al. discovered that periodontal ligament stem cells (PDLSCs)-derived exosomes alleviated the inflammatory microenvironment in chronic periodontitis *via* the Th17/Treg/miR-155-5p/SIRT1 regulatory network. EVs derived from LPS-pretreated PDLSCs induced M1 polarization of macrophages, whereas DNase I-treated EVs abolished M1 polarization. EVs derived from PDLSCs may be a potential therapeutic target for periodontal inflammation (189).

LPS-pretreated dental follicle stem cells (DFSCs)-sEVs polarized macrophages to an M2 phenotype through the ROS/ERK signaling pathway, inhibiting the alveolar bone loss and promoting periodontal regeneration in dogs with experimental periodontitis (69).

DCs-derived exosomes are relevant to immune therapy of periodontitis (190, 191). It has been demonstrated that engineered EVs derived from DCs can modulate the immune response in periodontitis and prevent inflammatory bone loss (192).

**Figure 2:**
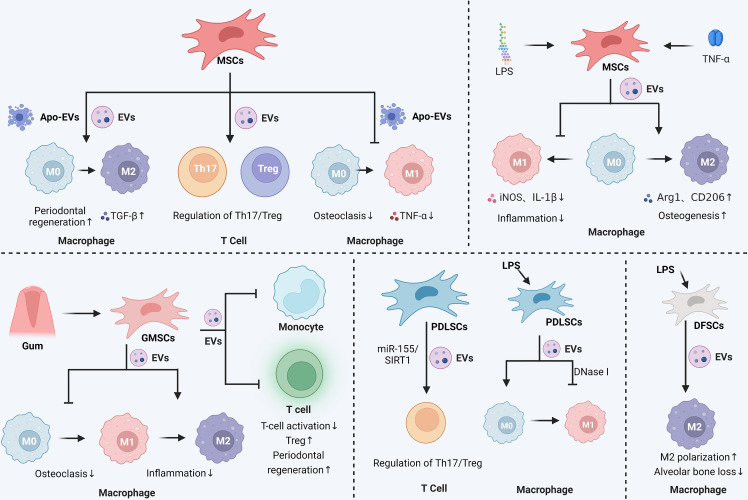
Immunomodulatory and anti-inflammatory effects of stem cell-derived EVs in periodontitis. In periodontitis, MSCs secreted EVs were able to promote the M2 polarization of macrophages and maintain the balance of Th17 and Treg cell ratio. MSCs secreted apoptotic vesicles were also able to inhibit the M1 polarization of macrophages, reduce the level of inflammatory mediators such as TNF-α, and promote the M2 polarization of macrophages. Pretreatment of MSCs with LPS or TNF-α enhanced the effect of MSCs-EVs, mainly by affecting macrophage polarization to control the inflammatory response and promote osteogenesis. Gingival mesenchymal stem cells (GMSCs) secrete EVs that affect macrophage polarization and inhibit osteoclastic and inflammatory reactions in periodontitis. GMSCs-EVs also inhibit monocyte and T-cell activation and promote periodontal tissue regeneration. Periodontal membrane stem cells (PDLSCs)-derived EVs could regulate the Th17/Treg ratio *via* the miR-155/SIRT1 axis and inhibit macrophage M1 polarization. LPS-stimulated dental follicular stem cells (DFSCs)-derived EVs also promoted macrophage M2 polarization and reduced alveolar bone loss. The Graph was created with BioRender.com. MSCs: mesenchymal stem cells; EVs, extracellular vesicles; Th, helper T cells; Treg, regulatory T cell; LPS, lipopolysaccharide.

Consequently, studies have presented that MSCs-EVs from various sources can promote M2 macrophage polarization, restrict osteoclast activity, and reduce alveolar bone resorption, which paves the way for the development of periodontitis therapy (**Figure 2**; **Table 2**).

**Table 2:** Anti-inflammatory and immunomodulatory effects of stem cell exosomes in periodontitis.

Author (Year)	MSCs source	Pretreatment of MSCs or EVs	Recipient of EVs	Experimental model	EVs administration	Functional outcome	Reference
Chew JRJ, et al., (70)	hMSC	/	rPDLSCs	①cells co-culture; ②Experimental periodontal defect rat;	①cells co-culture; ②Experimental periodontal defect rat: transplant/implant with exosome-loaded collagen sponge (CS/Exosome) or control collagen sponge (CS/Control);	repair periodontal defects, increase PDLSCs migration and proliferation	(67)
Cebatariuniene A, et al., (71)	hPDLSC	/	hPDLSCs	cells co-culture	cells co-culture	suppress basal and LPS‐induced activity of NFκB	(68)
Liu L, et al., (68)	rBMSC	/	hPDLSCs/RAW264.7 cells	①cells co-culture; ②Experimental Porphyromonas-induced periodontitis rats;	①cells co-culture; ②Experimental Porphyromonas-induced periodontitis rats: inject in periodontal pocket;	promote PDLSCs migration, proliferation and osteogenic differentiation	(65)
Huang Y, et al., (69)	hDFMSC	LPS pretreatment	hPDLSCs	①cells co-culture; ②Experimental Porphyromonas-induced periodontitis dogs;	①cells co-culture; ②Experimental Porphyromonas-induced periodontitis dogs: inject into the periodontal pocket;	promote PDLSCs proliferation and migration and macrophage proliferation	(66)
Kang M, et al., (180)	hBMSC	TNF-α pretreatment	mBMMs	①cells co-culture; ②Experimental calvaria defect rat;	①cells co-culture; ②Experimental calvaria defect rat: place on the wound by a clinical grade collagen scaffold (OraPLUG, Salvin);	immunoregulation, anti-inflammatory	(164)
Zhang Y, et al., (181)	hDPSC	3D culture	mouse naive CD4+ T cells	①cells co-culture; ②Experimental Ligature-induced periodontitis mice;	①cells co-culture; ②Experimental Ligature-induced periodontitis mice: inject into the palatal gingiva;	miR-1246 expression upregulated, reactive Th17 cell/Treg balance, anti-inflammatory	(165)
Xu R, et al., (182)	rBMSC	LPS pretreatment	Raw264.7 cells	①cells co-culture; ②Experimental myocardial infarction mice;	①cells co-culture; ②Experimental myocardial infarction mice: intramyocardial injection at four sites around the infarct border zone;	promote M2 macrophage polarization, attenuat the post-infarction inflammation and cardiomyocyte apoptosis	(166)
Ye Q, et al., (183)	mBMSC	/	mBMDMs	Porphyromonas gingivalis derived LPS (Pg-LPS) induced inflammation of mouse bone marrow-derived macrophages (mBMDMs)	cells co-culture	inhibit M1 macrophage polarization and TNF-α secretion	(167)
Yue C, et al., (184)	hBMSC	/	RAW264.7 cells	cells co-culture	cells co-culture	regulate macrophage metabolism, differentiation, and inflammation resolution	(168)
Nakao Y, et al., (185)	hGMSC	TNF-α pretreatment	hPBMCs	①cells co-culture; ②Experimental wound healing mice; ③Experimental Ligature-induced periodontitis mice;	①cells co-culture; ②Experimental wound healing mice: subcutaneously inject into the cutaneous wounds; ③Experimental Ligature-induced periodontitis mice: inject into the palatal gingiva of the ligated second maxillary molar;	promote M2 macrophage polarization, immunoregulation	(169)
Wang R, et al., (186)	hGMSC	/	THP-1 cells	cells co-culture	cells co-culture	promote M2 macrophage polarization, anti-inflammatory	(170)
Zarubova J, et al., (187)	hGMSC	/	macrophages	cells co-culture	cells co-culture	reactive Th17 cell/Treg balance, anti-inflammatory	(171)
Zheng Y, et al., (188)	hPDLSC	LPS pretreatment	CD4+ T cells	cells co-culture	cells co-culture	reactive Th17 cell/Treg balance, anti-inflammatory	(172)
Kang H, et al., (189)	hPDLSC	LPS pretreatment	THP-1 cells	cells co-culture	cells co-culture	inhibit M1 macrophage polarization and TNF-α secretion	(173)

### 5.2 Role of MSCs-EVs in periodontal regeneration

To achieve functional periodontal regeneration, periodontal ligament fibers need to be inserted between the newly produced cementum and alveolar bone (193). Recent studies are concerned mainly with the osteogenic and angiogenic properties of MSCs-EVs, which are critical elements of periodontal regeneration.

Zhu B (194) et al. co-cultured MSCs-Exo with PDLSCs and noticed increased proliferation and osteogenic differentiation of PDLSCs. Furthermore, *in vitro* experiments demonstrated that MSCs-Exo promoted the PDLSCs proliferation and migration by activating AKT and ERK signaling pathways (70). Hypoxic preconditioning of MSCs-sEVs significantly enhanced the proliferation, migration, and angiogenesis of human umbilical vein endothelial cells (UVECs) and promoted the formation of vascularized bone (32). In a rat model of the alveolar bone defect, Chew (70) et al. transplanted a collagen sponge loaded with MSCs-Exo and observed the regeneration of alveolar bone and functional periodontal ligament fibers.

Wei (195) et al. proposed that BMSCs-Exo derived from different stages of osteogenic induction could exert a sustained anti-inflammatory effect during osteogenesis, up-regulate genes associated with osteogenesis at the early stage, and promote MSCs migration at the later stage. EVs derived from neural EGFL-like 1 modified BMSCs were more capable of stimulating BMSCs osteogenesis due to the downregulation of miR-25-5p (196). Huang (197) et al. demonstrated that EVs derived from BMP2-overexpressing BMSCs preserved the essential physical and biochemical characteristics of BMSCs-EVs but showed greater bone regeneration capability in a rat calvarial defect model. The ApoEVs from dying BMSCs can effectively promote the viability of endogenous BMSCs and repair bone defects (198). In critical-size calvarial bone defects, BMSCs-EVs positively regulate osteogenic genes and osteoblast differentiation *in vitro* (199). It has also been reported that BMSCs-derived exosomes overexpressing hypoxia-inducible factor (HIF)-1α can increase the packaging of Jagged1 and angiogenesis of endothelial cells (ECs) via the Notch signaling pathway (200). BMSCs-derived Nidogen1-enriched EVs enhanced the migration and angiogenic capacity of rat arterial endothelial cells (AECs) and promoted bone regeneration in rat femoral defect models (201). Hui (202) et al. coated BMSCs-EVs on a demineralized bone matrix to create a functional scaffold with enhanced pro-angiogenic and pro-bone regeneration activities.

Through a rat periodontitis model, Mohammed (203) et al. found that the injection of adipose-derived mesenchymal stem cells (ADSCs) exosomes suspension can be used as an auxiliary tool to promote periodontal regeneration, specifically periodontal fibers, blood vessels, and alveolar bone. The polydopamine-coated poly(lactic-co-glycolic acid) (PLGA/pDA) scaffold combined with ADSCs-EVs significantly induced the alveolar bone defect repair in the rat model (204). The ADSCs exosomes immobilized on the PLGA/pDA scaffolds promote the repair of critical-size skull defects in rats by stimulating osteogenesis and promoting BMSCs migration and homing (205).

In the inflammatory microenvironment, dental pulp stem cells (DPSCs)-EVs may shutter LMBR1-targeting miR-758-5p via the BMP signaling pathway to promote osteogenic and odontogenic differentiation of PDLSCs and provide a potential strategy for bone regeneration (206). DPSCs exosomes can effectively reduce periodontal bone loss by stimulating the migration of human DPSCs and mouse osteoblasts (207). It is addressed that DPSCs-EVs can induce the regeneration of experimental bone defects by enhancing the phosphorylation of ERK 1/2 and JNK and promoting the osteogenic differentiation of ADSCs (208). Xian (209) et al. found that DPSCs exosomes could stimulate endothelial cell proliferation and pro-angiogenic factors production, such as FGF-2, VEGF-A, KDR, and MMP-9. It has been demonstrated that DPSCs-EVs isolated from periodontally healthy and unhealthy teeth can enhance endothelial cell angiogenesis activity, accelerate wound healing, and encourage angiogenesis in mouse skin lesions (210). The co-injection of DPSCs-EVs with collagen, β-tricalcium phosphate, or hydroxyapatite can stimulate new bone formation in rat skull defects (211).

Invitro studies performed by Wang (212) et al. demonstrated that stem cells of human exfoliated deciduous teeth (SHED)-Exo under osteogenic induction conditions could up-regulate the expressions of osteogenic markers like osteopontin (OPN), osteocalcin (OCN), and Runx2 during the osteogenic differentiation of PDLSCs and enhance the osteogenic differentiation of PDLSCs *via* Wnt and BMP signaling pathways. Wei (213) et al. observed that exosomes repaired the defect to the same extent as original stem cells, increased the osteogenic impact of BMSCs, and inhibited adipogenesis after injecting SHED-derived exosomes into the bone defect area of a mouse periodontitis model.

Purified PDLSCs-EVs were discovered to reduce LPS-induced NF-B activity in PDLSCs and enhance osteogenic mineralization in PDLSCs, which may be helpful for the targeted treatment of chronic inflammation in periodontitis (71). Engineered EVs from PDLSCs promoted bone regeneration and angiogenesis of skull defects in rats (214). Collagen membrane enriched with PDLSCs-EVs or polyethylenimine (PEI)-engineered EVs (PEI-EVs) can activate osteogenesis and promote bone regeneration (215). PDLSCs-EVs immobilized in matrigel accelerated bone tissue repair by inducing BMSCs proliferation and migration through increasing the phosphorylation of AKT and ERK1/2 (216).

GMSCs-exosomes were shown to contain a variety of growth factors, such as transforming growth factor-β (TGF-β) and vascular endothelial growth factor (VEGF), which were shown to promote pre-osteoblast migration and osteogenic differentiation (217). 3D-engineered scaffolds complexed with GMSCs-EVs exhibited strong osteogenic induction ability (218).

According to Ma L (219) et al., DFSCs-sEVs boosted PDLSC migration, proliferation, and osteogenic differentiation, which offers a novel approach to periodontal regeneration in the future.

**Figure 3:**
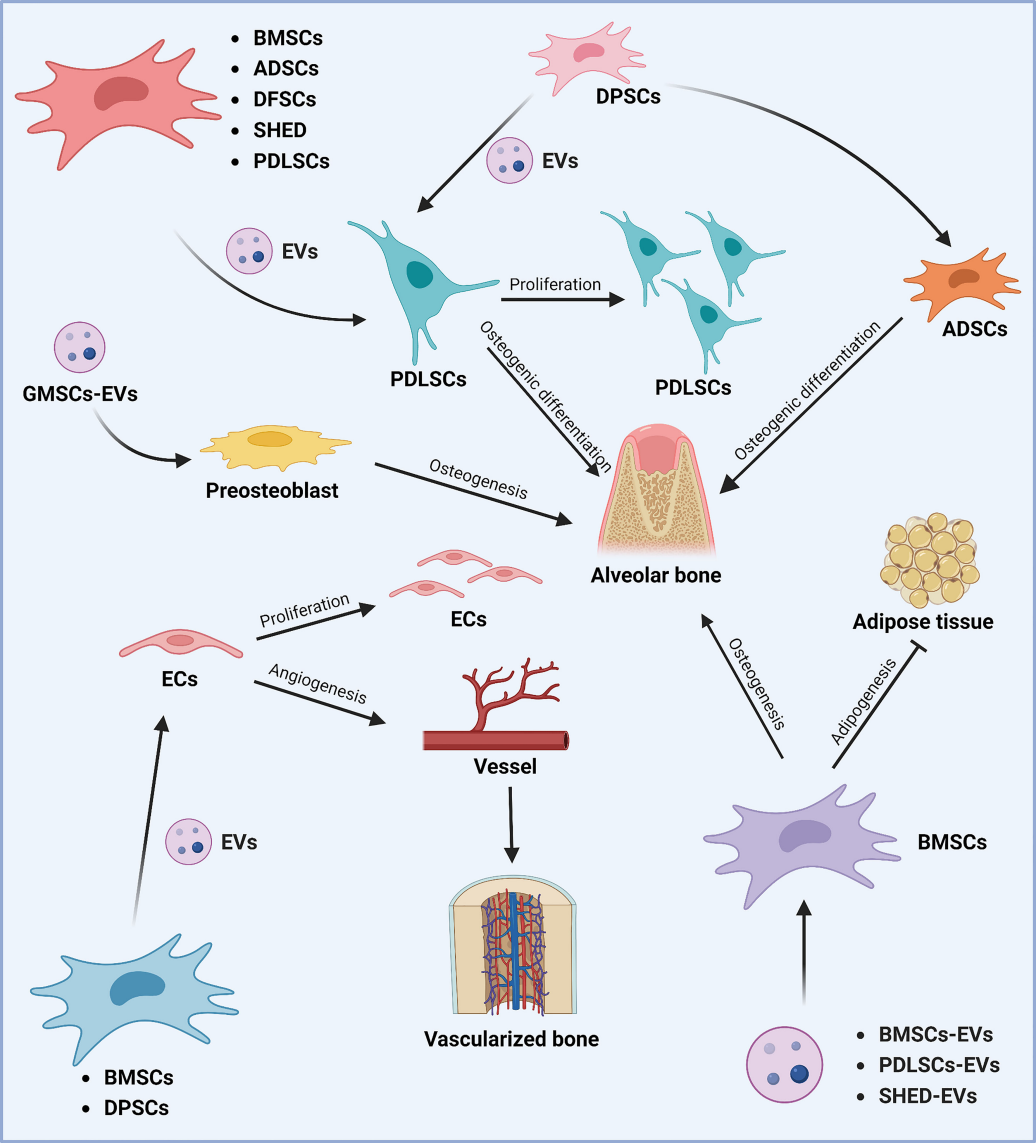
Role of stem cell-derived EVs in periodontal tissue regeneration. Different sources of MSCs can promote periodontal tissue regeneration and thus play a role in the treatment of periodontitis. Various sources of MSCs-EVs (including BMSCs, ADSCs, DFSCs, SHED, DPSCs, and PDLSCs) can promote the proliferation of periodontal membrane stem cells and alveolar bone osteogenesis by regulating osteogenic differentiation. DPSCs-derived EVs can promote alveolar bone regeneration by affecting the osteogenic differentiation of ADSCs. In addition, GMSCs-derived EVs can promote alveolar bone regeneration by regulating osteoblast precursor cells. In addition, BMSCs, PDLSCs, and SHED-derived EVs can promote osteogenic differentiation and inhibit lipogenic differentiation of BMSCs, thus promoting alveolar bone regeneration. The Graph was created with BioRender.com. BMSCs, bone marrow-derived mesenchymal stem cells; ADSCs, adipose tissue-derived mesenchymal stem cells; DFSCs, dental follicle stem cells; SHED, stem cells of human exfoliated deciduous teeth; PDLSCs, periodontal ligament stem cells; EVs, extracellular vesicles; GMSCs, gingiva-derived mesenchymal stem cells.

In conjunction with the current investigations, it is proposed that MSCs-EVs possess the potential to treat periodontitis and promote periodontal regeneration (**Figure 3**; **Table 3**). While the effectiveness of MSCs-EVs on periodontal ligament fibers and cementum regeneration needs to be further studied. Moreover, effectively alleviating the homeostasis imbalance is the key to periodontal regeneration. The application of MSCs-EVs in dentistry is restricted to fundamental research, and its clinical use requires more rigorous evidence. There is still a long way to go before MSCs-EVs can be used as an effective and safe dental clinical treatment method (220).

**Table 3:** Tissue regeneration of stem cell exosomes in periodontitis.

Author (Year)	MSCs source	Pretreatment of MSCs or EVs	Recipient of EVs	Experimental model	EVs administration	Functional outcome	Reference
Zhu B, et al. (194)	hPDLSC, hIBMMSC, JBMMSC	/	hPDLSCs	①cells co-culture; ②nude mice;	①cells co-culture; ②nude mice: transplant/implant into;	pro‐osteogenic, osteoimmunomodulatory	(178)
Zhuang Y, et al. (32)	hox-rBMSC	hypoxia pretreatment	HUVECs	①cells co-culture; ②Experimental calvarial defect rat;	①cells co-culture; ②Experimental calvarial defect rat: transplant/implant into;	promote HUVECs proliferation, migration and angiogenesis, pro‐osteogenic	(179)
Wei F, et al. (195)	hBMSC	/	hBMDMs and RAW264.7 cells(macrophages)	cells co-culture;	cells co-culture;	pro‐osteogenic, osteoimmunomodulatory	(180)
Lan Y, et al. (196)	rBMSC	neural EGFL-like 1 (Nell1) pretreatment	rBMSCs	①cells co-culture; ②Experimental calvarial defect rat;	①cells co-culture; ②Experimental calvarial defect rat: transplant/implant into;	pro‐osteogenic	(181)
Huang CC, et al. (197)	hBMSC	genetically modified by constitutively expressing BMP2(bone morphogenetic protein 2)	hBMSCs	①cells co-culture; ②Experimental calvarial defect rat;	①cells co-culture; ②Experimental calvarial defect rat: place on the wound;	pro‐osteogenic	(182)
Li M, et al. (198)	mBMSC	/	rBMSCs	①cells co-culture; ②Experimental calvarial defect rat;	①cells co-culture; ②Experimental calvarial defect rat: transplant/implant into;	promote rBMSCs proliferation, migration, and osteogenic differentiation	(183)
Qin Y, et al. (199)	hBMSC	/	human osteoblasts (hFOBs)	①cells co-culture; ②Experimental calvarial defect rat;	①cells co-culture; ②Experimental calvarial defect rat: transplant/implant into;	promote hFOBs proliferation, migration, and osteogenic differentiation	(184)
Gonzalez-King H, et al. (200)	hDPSC	hypoxia pretreatment	HUVECs	①cells co-culture; ②nude mice;	①cells co-culture; ②nude mice: inject subcutaneously into the flanks;	pro-angiogenic	(185)
Cheng P, et al. (201)	rBMSC	/	RAECs	①cells co-culture; ②nude mice;	①cells co-culture; ②nude mice: inject subcutaneously into the bac;	enhance RAECs migration, pro-angiogenic	(186)
Xie H, et al. (202)	rBMSC	/	HUVECs	①cells co-culture; ②nude mice;	①cells co-culture; ②nude mice: nude mice: implant into subcutaneously;	promote grafts vascularization, pro-angiogenic, pro‐osteogenic	(187)
Mohammed E, et al. (203)	hADSC	/	/	Experimental periodontal defect rat;	Experimental periodontal defect rat: inject in periodontal pocket;	immunomodulatory, anti-inflammatory, pro‐osteogenic	(188)
Yang Y, et al. (204)	hADSC	/	hPDLSCs	①cells co-culture; ②Experimental alveolar Bone Defects rat;	①cells co-culture; ②Experimental alveolar Bone Defects rat: transplant/implant into;	pro-angiogenic	(189)
Li W, et al. (205)	hADSC	/	hBMSCs	①cells co-culture; ②Experimental calvarial defect mice;	①cells co-culture; ②Experimental calvarial defect mice: transplant/implant into;	promote hBMSCs osteogenic, proliferation and migration, pro-angiogenic	(190)
Yan C, et al. (206)	ihDPSC	/	hPDLSCs	cells co-culture;	cells co-culture;	promote hPDLSCs osteogenic and osteogenic differentiation	(191)
Shimizu Y, et al. (207)	hDPSC	/	mouse osteoblastic MC3T3- E1 cells	①cells co-culture; ②Experimental periodontal defect mice;	①cells co-culture; ②Experimental periodontal defect mice: directly appliy onto the silk ligature;	promote MC3T3- E1 cells migration	(192)
Jin Q, et al. (208)	hDPSC	/	hADSCs	①cells co-culture; ②Experimental mandible defect rat;	①cells co-culture; ②Experimental mandible defect rat: injecte into the hydrogel scaffold material and sutur;	promoted hADSCs migration, mineralization and osteogenic differentiation	(193)
Xian X, et al. (209)	hDPSC	/	HUVECs	cells co-culture;	cells co-culture;	promote HUVECs proliferation and tube formation, pro-angiogenic	(194)
Zhou H, et al. (210)	hDPSC	/	ECs	①cells co-culture; ②Experimental skin wound healing mouse;	①cells co-culture; ②Experimental skin wound healing mouse: subcutaneously inject into the full-thickness excisional skin wound;	pro-angiogenic	(195)
Imanishi Y, et al. (211)	rDPSC	/	/	Experimental calvarial defect rat;	Experimental calvarial defect rat: transplant/implant into;	pro-angiogenic, pro‐osteogenic	(196)
Wang M, et al. (212)	hSHED	/	hPDLSCs	cells co-culture;	cells co-culture;	promote hPDLSCs osteogenic differentiation	(197)
Wei J, et al. (213)	hSHED	/	mBMSCs	①cells co-culture; ②Experimental periodontal defect rat;	①cells co-culture; ②Experimental periodontal defect rat: inject into the buccal and lingual sides of the first molar;	promote mBMSCs osteogenesis, differentiation, and bone formation	(198)
Pizzicannella J, et al. (214)	hPDLSC	/	hPDLSCs	①cells co-culture; ②Experimental calvarial defect rat;	①cells co-culture; ②Experimental calvarial defect rat: transplant/implant into;	pro-angiogenic, pro‐osteogenic	(199)
Diomede F, et al. (215)	hPDLSC	/	hPDLSCs	①cells co-culture; ②Experimental calvaria defect rat;	①cells co-culture; ②Experimental calvaria defect rat: transplant/implant into;	pro-angiogenic, pro‐osteogenic	(200)
Zhao B, et al. (216)	hPDLSC	/	hBMSCs	①cells co-culture; ②Experimental calvaria defect rat;	①cells co-culture: none; ②Experimental calvaria defect rat: transplant/implant into;	promote hBMSCs proliferation, migration, and osteogenic differentiation	(201)
Jiang S, et al. (217)	hPDLSC	/	mouse osteoblastic MC3T3- E1 cells	cells co-culture;	cells co-culture;	promote pre-osteoblasts migration and MC3T3-E1 cells osteogenic differentiation	(202)
Diomede F, et al. (218)	hGMSC	/	hGMSCs	①cells co-culture; ②Experimental calvaria defect rat;	①cells co-culture; ②Experimental calvaria defect rat: transplant/implant into;	pro‐osteogenic	(203)
Ma L, et al. (219)	hDFSC	/	hPDLSCs	①cells co-culture; ②Experimental periodontal defect rats;	①cells co-culture; ②Experimental periodontal defect rats: transplant/implant into;	promote hPDLSCs proliferation, migration, osteogenic differentiation	(204)

## 6 Summary and prospect

Several findings imply that OMVs can interfere with host gingival epithelium functions, affect angiogenesis, and induce PRRs reactions. OMVs also play a role in the pathogenicity of biofilms, such as promoting bacteria coaggregation, providing micronutrients to subgingival biofilms, and dispersing rival biofilms. These indicate that OMVs can promote connective tissue destruction and alveolar bone resorption. More research is required on the precise function and related mechanisms of OMVs in periodontitis. For example, the molecules and pathways by which OMVs affect innate and acquired immune defense (6).

Growing evidence suggests that saliva and GCF-derived EVs may serve as periodontitis biomarkers. Although the potential of saliva and GCF-derived EVs is promising, many obstacles must be solved before EVs can be translated into chair-side or off-the-shelf diagnostic tools. The major challenges are: (1) The need for standardized methods for EVs isolation and characterization. (2) More useful, sensitive, and specific biomarkers must be developed. (3) A large sample size analysis is required to establish the diagnostic standards for EVs-periodontitis that are appropriate for individuals of different ages and genders (23, 174). Despite the current challenges, the diagnostic potential of saliva and GCF-derived EVs is compelling, and future clinical applications can be expected.

MSCs-derived EVs have massive advantages, particularly convenient access, a wide range of sources, immunomodulatory ability, and tissue repair and regeneration capacities. EVs derived from MSCs have emerged over the past decades as an alternative therapy for stem cells in the field of regenerative medicine (221), and they are expected to be a novel therapeutic tool for periodontal regeneration (222). However, the oral cavity is a highly complex environment constantly changing, and it is unclear how several elements, including temperature, pH, oxygen, inflammation, and microbiota species, affect EVs. The majority of current MSCs-EVs studies use animal models. Yet the clinical application of MSCs-EVs in periodontal regeneration has not been reported. There are no standardized methods for the clinically graded manufacture and quality control of EVs medicines, which are crucial in following EVs clinical trials (223). In addition, there are still no low-cost technologies to swiftly produce an abundance of highly homogenous MSCs-EVs (224).

It is anticipated that more comprehensive research on EVs affecting periodontitis will be produced, demonstrating tremendous promise for clinical treatment, and opening up new doors for the advancement of stomatology.

## Author contributions

CM and JP were responsible for constructing the concept of the paper. JP and WZ contributed to the literature search and analysis. CM, RC, and LW were responsible for writing the manuscript draft. BL and YW participated in editing and finalizing the manuscript. All authors have agreed with the final version of the manuscript before submission. All authors read and approved the final manuscript. All authors contributed to the article and approved the submitted version.

## References

[1] Hasturk H Schulte F Martins M Sherzai H Floros C Cugini M. Safety and preliminary efficacy of a novel host-modulatory therapy for reducing gingival inflammation. Front Immunol (2021) 12:704163. doi: 10.3389/fimmu.2021.704163 34589083 PMC8475270

[2] Graves DT Cochran D. The contribution of interleukin-1 and tumor necrosis factor to periodontal tissue destruction. J Periodontol (2003) 74(3):391–401. doi: 10.1902/jop.2003.74.3.391 12710761

[3] Collaborators GBDOD Bernabe E Marcenes W Hernandez CR Bailey J Abreu LG. Global, regional, and national levels and trends in burden of oral conditions from 1990 to 2017: A systematic analysis for the global burden of disease 2017 study. J Dent Res (2020) 99(4):362–73. doi: 10.1177/0022034520908533 PMC7088322 32122215

[4] Peres MA Macpherson LMD Weyant RJ Daly B Venturelli R Mathur MR. Oral diseases: a global public health challenge. Lancet. (2019) 394(10194):249–60. doi: 10.1016/S0140-6736(19)31146-8 31327369

[5] Chapple IL. Time to take periodontitis seriously. BMJ (2014) 348:g2645. doi: 10.1136/bmj.g2645 24721751

[6] Yamamoto M Aizawa R. Maintaining a protective state for human periodontal tissue. Periodontol 2000. (2021) 86(1):142–56. doi: 10.1111/prd.12367 33690927

[7] Moutsopoulos NM Konkel JE. Tissue-specific immunity at the oral mucosal barrier. Trends Immunol (2018) 39(4):276–87. doi: 10.1016/j.it.2017.08.005 PMC5843496 28923364

[8] Van Dyke TE. The management of inflammation in periodontal disease. J Periodontol (2008) 79(8 Suppl):1601–8. doi: 10.1902/jop.2008.080173 PMC2563957 18673016

[9] Okada H Murakami S. Cytokine expression in periodontal health and disease. Crit Rev Oral Biol Med (1998) 9(3):248–66. doi: 10.1177/10454411980090030101 9715365

[10] Page RC Offenbacher S Schroeder HE Seymour GJ Kornman KS. Advances in the pathogenesis of periodontitis: summary of developments, clinical implications and future directions. Periodontol 2000. (1997) 14:216–48. doi: 10.1111/j.1600-0757.1997.tb00199.x 9567973

[11] Hajishengallis E Hajishengallis G. Neutrophil homeostasis and periodontal health in children and adults. J Dent Res (2014) 93(3):231–7. doi: 10.1177/0022034513507956 PMC3929973 24097856

[12] Chakravarti A Raquil MA Tessier P Poubelle PE. Surface RANKL of toll-like receptor 4-stimulated human neutrophils activates osteoclastic bone resorption. Blood. (2009) 114(8):1633–44. doi: 10.1182/blood-2008-09-178301 19546479

[13] Graves D. Cytokines that promote periodontal tissue destruction. J Periodontol (2008) 79(8 Suppl):1585–91. doi: 10.1902/jop.2008.080183 18673014

[14] Lamont RJ Koo H Hajishengallis G. The oral microbiota: dynamic communities and host interactions. Nat Rev Microbiol (2018) 16(12):745–59. doi: 10.1038/s41579-018-0089-x PMC6278837 30301974

[15] Hajishengallis G. New developments in neutrophil biology and periodontitis. Periodontol 2000. (2020) 82(1):78–92. doi: 10.1111/prd.12313 31850633

[16] Pan W Wang Q Chen Q. The cytokine network involved in the host immune response to periodontitis. Int J Oral Sci (2019) 11(3):30. doi: 10.1038/s41368-019-0064-z 31685798 PMC6828663

[17] Kinane DF. Causation and pathogenesis of periodontal disease. Periodontol 2000 (2001) 25:8–20. doi: 10.1034/j.1600-0757.2001.22250102.x 11155179

[18] American Academy of periodontology task force report on the update to the 1999 classification of periodontal diseases and conditions. J Periodontol (2015) 86(7):835–8. doi: 10.1902/jop.2015.157001 26125117

[19] Haffajee AD Socransky SS Goodson JM. Clinical parameters as predictors of destructive periodontal disease activity. J Clin Periodontol (1983) 10(3):257–65. doi: 10.1111/j.1600-051x.1983.tb01274.x 6575980

[20] Renvert S Persson GR. A systematic review on the use of residual probing depth, bleeding on probing and furcation status following initial periodontal therapy to predict further attachment and tooth loss. J Clin Periodontol (2002) 29 Suppl 3:82–9. doi: 10.1034/j.1600-051x.29.s-3.2.x 12787209

[21] Chapple IL. Periodontal disease diagnosis: current status and future developments. J Dent. (1997) 25(1):3–15. doi: 10.1016/s0300-5712(95)00118-2 9080734

[22] Marini L Tonetti MS Nibali L Rojas MA Aimetti M Cairo F. The staging and grading system in defining periodontitis cases: consistency and accuracy amongst periodontal experts, general dentists and undergraduate students. J Clin Periodontol (2021) 48(2):205–15. doi: 10.1111/jcpe.13406 33260273

[23] Han P Bartold PM Ivanovski S. The emerging role of small extracellular vesicles in saliva and gingival crevicular fluid as diagnostics for periodontitis. J Periodontal Res (2022) 57(1):219–31. doi: 10.1111/jre.12950 34773636

[24] Armitage GC. The complete periodontal examination. Periodontol 2000 (2004) 34:22–33. doi: 10.1046/j.0906-6713.2002.003422.x 14717853

[25] Khattri S Kumbargere Nagraj S Arora A Eachempati P Kusum CK Bhat KG. Adjunctive systemic antimicrobials for the non-surgical treatment of periodontitis. Cochrane Database Syst Rev (2020) 11(11):CD012568. doi: 10.1002/14651858.CD012568.pub2. 33197289 PMC9166531

[26] Cobb CM. Clinical significance of non-surgical periodontal therapy: an evidence-based perspective of scaling and root planing. J Clin Periodontol (2002) 29(Suppl 2):6–16. doi: 10.1034/j.1600-051X.29.s2.2.x 12010523

[27] Graziani F Karapetsa D Alonso B Herrera D. Nonsurgical and surgical treatment of periodontitis: how many options for one disease? Periodontol 2000 (2017) 75(1):152–88. doi: 10.1111/prd.12201. 28758300

[28] Liu J Ruan J Weir MD Ren K Schneider A Wang P. Periodontal bone-Ligament-Cementum regeneration via scaffolds and stem cells. Cells (2019) 8(6):537. doi: 10.3390/cells8060537 31167434 PMC6628570

[29] Wang HL Greenwell H. Surgical periodontal therapy. Periodontol 2000 (2001) 25:89–99. doi: 10.1034/j.1600-0757.2001.22250107.x 11155184

[30] Han J Menicanin D Gronthos S Bartold PM. Stem cells, tissue engineering and periodontal regeneration. Aust Dent J (2014) 59 Suppl 1:117–30. doi: 10.1111/adj.12100 24111843

[31] Ding T Kang W Li J Yu L Ge S. An *in situ* tissue engineering scaffold with growth factors combining angiogenesis and osteoimmunomodulatory functions for advanced periodontal bone regeneration. J Nanobiotechnology. (2021) 19(1):247. doi: 10.1186/s12951-021-00992-4 34404409 PMC8371786

[32] Zhuang Y Cheng M Li M Cui J Huang J Zhang C. Small extracellular vesicles derived from hypoxic mesenchymal stem cells promote vascularized bone regeneration through the miR-210-3p/EFNA3/PI3K pathway. Acta Biomater. (2022) 15(150):150:413–426. doi: 10.1016/j.actbio.2022.07.015 35850484

[33] Nyman S Lindhe J Karring T Rylander H. New attachment following surgical treatment of human periodontal disease. J Clin Periodontol (1982) 9(4):290–6. doi: 10.1111/j.1600-051x.1982.tb02095.x 6964676

[34] Larsson L Decker AM Nibali L Pilipchuk SP Berglundh T Giannobile WV. Regenerative medicine for periodontal and peri-implant diseases. J Dent Res (2016) 95(3):255–66. doi: 10.1177/0022034515618887 PMC4766955 26608580

[35] Chen FM Zhang J Zhang M An Y Chen F Wu ZF. A review on endogenous regenerative technology in periodontal regenerative medicine. Biomaterials. (2010) 31(31):7892–927. doi: 10.1016/j.biomaterials.2010.07.019 20684986

[36] Nibali L Koidou VP Nieri M Barbato L Pagliaro U Cairo F. Regenerative surgery versus access flap for the treatment of intra-bony periodontal defects: A systematic review and meta-analysis. J Clin Periodontol (2020) 47 Suppl 22:320–51. doi: 10.1111/jcpe.13237 31860134

[37] Sculean A Nikolidakis D Nikou G Ivanovic A Chapple IL Stavropoulos A. Biomaterials for promoting periodontal regeneration in human intrabony defects: a systematic review. Periodontol 2000. (2015) 68(1):182–216. doi: 10.1111/prd.12086 25867987

[38] Qin Y Sun R Wu C Wang L Zhang C. Exosome: A novel approach to stimulate bone regeneration through regulation of osteogenesis and angiogenesis. Int J Mol Sci (2016) 17(5):712. doi: 10.3390/ijms17050712. 27213355 PMC4881534

[39] van Niel G D'Angelo G Raposo G. Shedding light on the cell biology of extracellular vesicles. Nat Rev Mol Cell Biol (2018) 19(4):213–28. doi: 10.1038/nrm.2017.125 29339798

[40] Schuh C Cuenca J Alcayaga-Miranda F Khoury M. Exosomes on the border of species and kingdom intercommunication. Transl Res (2019) 210:80–98. doi: 10.1016/j.trsl.2019.03.008 30998903

[41] Doyle LM Wang MZ. Overview of extracellular vesicles, their origin, composition, purpose, and methods for exosome isolation and analysis. Cells (2019) 8(7):727. doi: 10.3390/cells8070727. 31311206 PMC6678302

[42] Zhang X Zhang H Gu J Zhang J Shi H Qian H. Engineered extracellular vesicles for cancer therapy. Adv Mater (2021) 33(14):e2005709. doi: 10.1002/adma.202005709 33644908

[43] Waldenstrom A Ronquist G. Role of exosomes in myocardial remodeling. Circ Res (2014) 114(2):315–24. doi: 10.1161/CIRCRESAHA.114.300584 24436427

[44] Villarroya-Beltri C Baixauli F Mittelbrunn M Fernandez-Delgado I Torralba D Moreno-Gonzalo O. ISGylation controls exosome secretion by promoting lysosomal degradation of MVB proteins. Nat Commun (2016) 7:13588. doi: 10.1038/ncomms13588 27882925 PMC5123068

[45] Akers JC Gonda D Kim R Carter BS Chen CC. Biogenesis of extracellular vesicles (EV): exosomes, microvesicles, retrovirus-like vesicles, and apoptotic bodies. J Neurooncol. (2013) 113(1):1–11. doi: 10.1007/s11060-013-1084-8 23456661 PMC5533094

[46] Tkach M Thery C. Communication by extracellular vesicles: Where we are and where we need to go. Cell. (2016) 164(6):1226–32. doi: 10.1016/j.cell.2016.01.043 26967288

[47] Thery C Witwer KW Aikawa E Alcaraz MJ Anderson JD Andriantsitohaina R. Minimal information for studies of extracellular vesicles 2018 (MISEV2018): a position statement of the international society for extracellular vesicles and update of the MISEV2014 guidelines. J Extracell Vesicles. (2018) 7(1):1535750. doi: 10.1080/20013078.2018.1535750 30637094 PMC6322352

[48] Mulcahy LA Pink RC Carter DR. Routes and mechanisms of extracellular vesicle uptake. J Extracell Vesicles. (2014) 3(1):24641. doi: 10.3402/jev.v3.24641. PMC4122821 25143819

[49] Walker S Busatto S Pham A Tian M Suh A Carson K. Extracellular vesicle-based drug delivery systems for cancer treatment. Theranostics. (2019) 9(26):8001–17. doi: 10.7150/thno.37097 PMC6857056 31754377

[50] Huang CC Kang M Narayanan R DiPietro LA Cooper LF Gajendrareddy P. Evaluating the endocytosis and lineage-specification properties of mesenchymal stem cell derived extracellular vesicles for targeted therapeutic applications. Front Pharmacol (2020) 11:163. doi: 10.3389/fphar.2020.00163 32194405 PMC7063066

[51] Pan BT Johnstone RM. Fate of the transferrin receptor during maturation of sheep reticulocytes *in vitro*: selective externalization of the receptor. Cell. (1983) 33(3):967–78. doi: 10.1016/0092-8674(83)90040-5 6307529

[52] Huang T Song C Zheng L Xia L Li Y Zhou Y. The roles of extracellular vesicles in gastric cancer development, microenvironment, anti-cancer drug resistance, and therapy. Mol Cancer. (2019) 18(1):62. doi: 10.1186/s12943-019-0967-5 30925929 PMC6441168

[53] Wiklander OPB Brennan MA Lotvall J Breakefield XO El Andaloussi S. Advances in therapeutic applications of extracellular vesicles. Sci Transl Med (2019) 11(492):eaav8521. doi: 10.1126/scitranslmed.aav8521. 31092696 PMC7104415

[54] Kalluri R LeBleu VS. The biology, function, and biomedical applications of exosomes. Science (2020) 367(6478):eaau6977. doi: 10.1126/science.aau6977. 32029601 PMC7717626

[55] Momen-Heravi F Getting SJ Moschos SA. Extracellular vesicles and their nucleic acids for biomarker discovery. Pharmacol Ther (2018) 192:170–87. doi: 10.1016/j.pharmthera.2018.08.002 30081050

[56] Lasser C Jang SC Lotvall J. Subpopulations of extracellular vesicles and their therapeutic potential. Mol Aspects Med (2018) 60:1–14. doi: 10.1016/j.mam.2018.02.002 29432782

[57] Phinney DG Pittenger MF. Concise review: MSC-derived exosomes for cell-free therapy. Stem Cells (2017) 35(4):851–8. doi: 10.1002/stem.2575 28294454

[58] Saint-Pol J Gosselet F Duban-Deweer S Pottiez G Karamanos Y. Targeting and crossing the blood-brain barrier with extracellular vesicles. Cells (2020) 9(4):851. doi: 10.3390/cells9040851 32244730 PMC7226770

[59] Hu C Zhao L Zhang L Bao Q Li L. Mesenchymal stem cell-based cell-free strategies: safe and effective treatments for liver injury. Stem Cell Res Ther (2020) 11(1):377. doi: 10.1186/s13287-020-01895-1 32883343 PMC7469278

[60] Hade MD Suire CN Mossell J Suo Z. Extracellular vesicles: Emerging frontiers in wound healing. Med Res Rev (2022) 42(6):2102–25. doi: 10.1002/med.21918 35757979

[61] Sohrabi B Dayeri B Zahedi E Khoshbakht S Nezamabadi Pour N Ranjbar H. Mesenchymal stem cell (MSC)-derived exosomes as novel vehicles for delivery of miRNAs in cancer therapy. Cancer Gene Ther (2022) 29(8-9):1105–16. doi: 10.1038/s41417-022-00427-8 35082400

[62] Huang YC Lai LC. The potential roles of stem cell-derived extracellular vesicles as a therapeutic tool. Ann Transl Med (2019) 7(22):693. doi: 10.21037/atm.2019.11.66 31930094 PMC6944607

[63] Ha DH Kim SD Lee J Kwon HH Park GH Yang SH. Toxicological evaluation of exosomes derived from human adipose tissue-derived mesenchymal stem/stromal cells. Regul Toxicol Pharmacol (2020) 115:104686. doi: 10.1016/j.yrtph.2020.104686 32450131

[64] Spees JL Lee RH Gregory CA. Mechanisms of mesenchymal stem/stromal cell function. Stem Cell Res Ther (2016) 7(1):125. doi: 10.1186/s13287-016-0363-7 27581859 PMC5007684

[65] Krampera M Le Blanc K. Mesenchymal stromal cells: Putative microenvironmental modulators become cell therapy. Cell Stem Cell (2021) 28(10):1708–25. doi: 10.1016/j.stem.2021.09.006 34624232

[66] Lei F Li M Lin T Zhou H Wang F Su X. Treatment of inflammatory bone loss in periodontitis by stem cell-derived exosomes. Acta Biomater. (2022) 141:333–43. doi: 10.1016/j.actbio.2021.12.035 34979326

[67] Jiang X Lew KS Chen Q Richards AM Wang P. Human mesenchymal stem cell-derived exosomes reduce Ischemia/Reperfusion injury by the inhibitions of apoptosis and autophagy. Curr Pharm Des (2018) 24(44):5334–41. doi: 10.2174/1381612825666190119130441 30659531

[68] Liu L Guo S Shi W Liu Q Huo F Wu Y. Bone marrow mesenchymal stem cell-derived small extracellular vesicles promote periodontal regeneration. Tissue Eng Part A. (2021) 27(13-14):962–76. doi: 10.1089/ten.TEA.2020.0141 32962564

[69] Huang Y Liu Q Liu L Huo F Guo S Tian W. Lipopolysaccharide-preconditioned dental follicle stem cells derived small extracellular vesicles treating periodontitis *via* reactive oxygen Species/Mitogen-activated protein kinase signaling-mediated antioxidant effect. Int J Nanomedicine. (2022) 17:799–819. doi: 10.2147/IJN.S350869 35228798 PMC8882029

[70] Chew JRJ Chuah SJ Teo KYW Zhang S Lai RC Fu JH. Mesenchymal stem cell exosomes enhance periodontal ligament cell functions and promote periodontal regeneration. Acta Biomater. (2019) 89:252–64. doi: 10.1016/j.actbio.2019.03.021 30878447

[71] Cebatariuniene A Kriauciunaite K Prunskaite J Tunaitis V Pivoriunas A. Extracellular vesicles suppress basal and lipopolysaccharide-induced NFkappaB activity in human periodontal ligament stem cells. Stem Cells Dev (2019) 28(15):1037–49. doi: 10.1089/scd.2019.0021 31017040

[72] Veerman RE Teeuwen L Czarnewski P Gucluler Akpinar G Sandberg A Cao X. Molecular evaluation of five different isolation methods for extracellular vesicles reveals different clinical applicability and subcellular origin. J Extracell Vesicles. (2021) 10(9):e12128. doi: 10.1002/jev2.12128 34322205 PMC8298890

[73] Watson DC Yung BC Bergamaschi C Chowdhury B Bear J Stellas D. Scalable, cGMP-compatible purification of extracellular vesicles carrying bioactive human heterodimeric IL-15/lactadherin complexes. J Extracell Vesicles. (2018) 7(1):1442088. doi: 10.1080/20013078.2018.1442088 29535850 PMC5844027

[74] Roda B Zattoni A Reschiglian P Moon MH Mirasoli M Michelini E. Field-flow fractionation in bioanalysis: A review of recent trends. Anal Chim Acta (2009) 635(2):132–43. doi: 10.1016/j.aca.2009.01.015 19216870

[75] Heath N Grant L De Oliveira TM Rowlinson R Osteikoetxea X Dekker N. Rapid isolation and enrichment of extracellular vesicle preparations using anion exchange chromatography. Sci Rep (2018) 8(1):5730. doi: 10.1038/s41598-018-24163-y 29636530 PMC5893571

[76] Liu C Guo J Tian F Yang N Yan F Ding Y. Field-free isolation of exosomes from extracellular vesicles by microfluidic viscoelastic flows. ACS Nano. (2017) 11(7):6968–76. doi: 10.1021/acsnano.7b02277 28679045

[77] Lewis JM Vyas AD Qiu Y Messer KS White R Heller MJ. Integrated analysis of exosomal protein biomarkers on alternating current electrokinetic chips enables rapid detection of pancreatic cancer in patient blood. ACS Nano. (2018) 12(4):3311–20. doi: 10.1021/acsnano.7b08199 29570265

[78] Lee K Shao H Weissleder R Lee H. Acoustic purification of extracellular microvesicles. ACS Nano. (2015) 9(3):2321–7. doi: 10.1021/nn506538f PMC4373978 25672598

[79] Merchant ML Powell DW Wilkey DW Cummins TD Deegens JK Rood IM. Microfiltration isolation of human urinary exosomes for characterization by MS. Proteomics Clin Appl (2010) 4(1):84–96. doi: 10.1002/prca.200800093 21137018

[80] Higginbotham JN Zhang Q Jeppesen DK Scott AM Manning HC Ochieng J. Identification and characterization of EGF receptor in individual exosomes by fluorescence-activated vesicle sorting. J Extracell Vesicles. (2016) 5:29254. doi: 10.3402/jev.v5.29254 27345057 PMC4921784

[81] Mallardi A Nuzziello N Liguori M Avolio C Palazzo G. Counting of peripheral extracellular vesicles in multiple sclerosis patients by an improved nanoplasmonic assay and dynamic light scattering. Colloids Surf B Biointerfaces. (2018) 168:134–42. doi: 10.1016/j.colsurfb.2018.02.006 29428682

[82] van der Pol E Coumans FA Grootemaat AE Gardiner C Sargent IL Harrison P. Nieuwland r. particle size distribution of exosomes and microvesicles determined by transmission electron microscopy, flow cytometry, nanoparticle tracking analysis, and resistive pulse sensing. J Thromb Haemost. (2014) 12(7):1182–92. doi: 10.1111/jth.12602 24818656

[83] Kotrbova A Stepka K Maska M Palenik JJ Ilkovics L Klemova D. TEM ExosomeAnalyzer: a computer-assisted software tool for quantitative evaluation of extracellular vesicles in transmission electron microscopy images. J Extracell Vesicles. (2019) 8(1):1560808. doi: 10.1080/20013078.2018.1560808 30719239 PMC6346710

[84] Zhao M Zhang M Xu K Wu K Xie R Li R. Antimicrobial effect of extracellular vesicles derived from human oral mucosal epithelial cells on candida albicans. Front Immunol (2022) 13:777613. doi: 10.3389/fimmu.2022.777613 35844569 PMC9283572

[85] Kim SY Khanal D Kalionis B Chrzanowski W. High-fidelity probing of the structure and heterogeneity of extracellular vesicles by resonance-enhanced atomic force microscopy infrared spectroscopy. Nat Protoc (2019) 14(2):576–93. doi: 10.1038/s41596-018-0109-3 30651586

[86] Stec A Jonca J Waleron K Waleron M Ploska A Kalinowski L. Quality control of bacterial extracellular vesicles with total protein content assay, nanoparticles tracking analysis, and capillary electrophoresis. Int J Mol Sci (2022) 23(8):4347. doi: 10.3390/ijms23084347 35457164 PMC9028362

[87] Corso G Heusermann W Trojer D Gorgens A Steib E Voshol J. Systematic characterization of extracellular vesicle sorting domains and quantification at the single molecule - single vesicle level by fluorescence correlation spectroscopy and single particle imaging. J Extracell Vesicles. (2019) 8(1):1663043. doi: 10.1080/20013078.2019.1663043 31579435 PMC6758720

[88] Chudzik A Migdal P Pasciak M. Different cutibacterium acnes phylotypes release distinct extracellular vesicles. Int J Mol Sci (2022) 23(10):5797. doi: 10.3390/ijms23105797. 35628607 PMC9147970

[89] Crescitelli R Lasser C Lotvall J. Isolation and characterization of extracellular vesicle subpopulations from tissues. Nat Protoc (2021) 16(3):1548–80. doi: 10.1038/s41596-020-00466-1 33495626

[90] Duijvesz D Versluis CY van der Fels CA Vredenbregt-van den Berg MS Leivo J Peltola MT. Immuno-based detection of extracellular vesicles in urine as diagnostic marker for prostate cancer. Int J Cancer. (2015) 137(12):2869–78. doi: 10.1002/ijc.29664 26139298

[91] Suarez H Gamez-Valero A Reyes R Lopez-Martin S Rodriguez MJ Carrascosa JL. A bead-assisted flow cytometry method for the semi-quantitative analysis of extracellular vesicles. Sci Rep (2017) 7(1):11271. doi: 10.1038/s41598-017-11249-2 28900146 PMC5595788

[92] Xia Y Liu M Wang L Yan A He W Chen M. And colorimetric aptasensor based on DNA-capped single-walled carbon nanotubes for detection of exosomes. Biosens Bioelectron. (2017) 92:8–15. doi: 10.1016/j.bios.2017.01.063 28167415

[93] Rupert DL Lasser C Eldh M Block S Zhdanov VP Lotvall JO. Determination of exosome concentration in solution using surface plasmon resonance spectroscopy. Anal Chem (2014) 86(12):5929–36. doi: 10.1021/ac500931f 24848946

[94] Mombelli A. Microbial colonization of the periodontal pocket and its significance for periodontal therapy. Periodontol 2000. (2018) 76(1):85–96. doi: 10.1111/prd.12147 29193304

[95] Moore WE Moore LH Ranney RR Smibert RM Burmeister JA Schenkein HA. The microflora of periodontal sites showing active destructive progression. J Clin Periodontol (1991) 18(10):729–39. doi: 10.1111/j.1600-051x.1991.tb00064.x 1752997

[96] Bartold PM Van Dyke TE. An appraisal of the role of specific bacteria in the initial pathogenesis of periodontitis. J Clin Periodontol (2019) 46(1):6–11. doi: 10.1111/jcpe.13046 PMC6357965 30556922

[97] Gong T Chen Q Mao H Zhang Y Ren H Xu M. Outer membrane vesicles of porphyromonas gingivalis trigger NLRP3 inflammasome and induce neuroinflammation, tau phosphorylation, and memory dysfunction in mice. Front Cell Infect Microbiol (2022) 12:925435. doi: 10.3389/fcimb.2022.925435 36017373 PMC9397999

[98] Seyama M Yoshida K Yoshida K Fujiwara N Ono K Eguchi T. Outer membrane vesicles of porphyromonas gingivalis attenuate insulin sensitivity by delivering gingipains to the liver. Biochim Biophys Acta Mol Basis Dis (2020) 1866(6):165731. doi: 10.1016/j.bbadis.2020.165731 32088316

[99] Darveau RP Hajishengallis G Curtis MA. Porphyromonas gingivalis as a potential community activist for disease. J Dent Res (2012) 91(9):816–20. doi: 10.1177/0022034512453589 PMC3420389 22772362

[100] Cecil JD O'Brien-Simpson NM Lenzo JC Holden JA Singleton W Perez-Gonzalez A. Outer membrane vesicles prime and activate macrophage inflammasomes and cytokine secretion *In vitro* and in vivo. Front Immunol (2017) 8:1017. doi: 10.3389/fimmu.2017.01017 28890719 PMC5574916

[101] Sartorio MG Pardue EJ Feldman MF Haurat MF. Bacterial outer membrane vesicles: From discovery to applications. Annu Rev Microbiol (2021) 75:609–30. doi: 10.1146/annurev-micro-052821-031444 PMC8500939 34351789

[102] Toyofuku M Nomura N Eberl L. Types and origins of bacterial membrane vesicles. Nat Rev Microbiol (2019) 17(1):13–24. doi: 10.1038/s41579-018-0112-2 30397270

[103] Schwechheimer C Kuehn MJ. Outer-membrane vesicles from gram-negative bacteria: biogenesis and functions. Nat Rev Microbiol (2015) 13(10):605–19. doi: 10.1038/nrmicro3525 PMC5308417 26373371

[104] Ma L Cao Z. Membrane vesicles from periodontal pathogens and their potential roles in periodontal disease and systemic illnesses. J Periodontal Res (2021) 56(4):646–55. doi: 10.1111/jre.12884 33826135

[105] Meyle J Chapple I. Molecular aspects of the pathogenesis of periodontitis. Periodontol 2000. (2015) 69(1):7–17. doi: 10.1111/prd.12104 26252398

[106] Kuehn MJ Kesty NC. Bacterial outer membrane vesicles and the host-pathogen interaction. Genes Dev (2005) 19(22):2645–55. doi: 10.1101/gad.1299905 16291643

[107] Haurat MF Aduse-Opoku J Rangarajan M Dorobantu L Gray MR Curtis MA. Selective sorting of cargo proteins into bacterial membrane vesicles. J Biol Chem (2011) 286(2):1269–76. doi: 10.1074/jbc.M110.185744 PMC3020734 21056982

[108] Bomberger JM Maceachran DP Coutermarsh BA Ye S O'Toole GA Stanton BA. Long-distance delivery of bacterial virulence factors by pseudomonas aeruginosa outer membrane vesicles. PloS Pathog (2009) 5(4):e1000382. doi: 10.1371/journal.ppat.1000382 19360133 PMC2661024

[109] Weiner A Mellouk N Lopez-Montero N Chang YY Souque C Schmitt C. Macropinosomes are key players in early shigella invasion and vacuolar escape in epithelial cells. PloS Pathog (2016) 12(5):e1005602. doi: 10.1371/journal.ppat.1005602 27182929 PMC4868309

[110] Canas MA Gimenez R Fabrega MJ Toloza L Baldoma L Badia J. Outer membrane vesicles from the probiotic escherichia coli nissle 1917 and the commensal ECOR12 enter intestinal epithelial cells *via* clathrin-dependent endocytosis and elicit differential effects on DNA damage. PloS One (2016) 11(8):e0160374. doi: 10.1371/journal.pone.0160374 27487076 PMC4972321

[111] Nakao R Takashiba S Kosono S Yoshida M Watanabe H Ohnishi M. Effect of porphyromonas gingivalis outer membrane vesicles on gingipain-mediated detachment of cultured oral epithelial cells and immune responses. Microbes Infect (2014) 16(1):6–16. doi: 10.1016/j.micinf.2013.10.005 24140554

[112] Cecil JD O'Brien-Simpson NM Lenzo JC Holden JA Chen YY Singleton W. Differential responses of pattern recognition receptors to outer membrane vesicles of three periodontal pathogens. PloS One (2016) 11(4):e0151967. doi: 10.1371/journal.pone.0151967 27035339 PMC4818014

[113] Bonnington KE Kuehn MJ. Protein selection and export *via* outer membrane vesicles. Biochim Biophys Acta (2014) 1843(8):1612–9. doi: 10.1016/j.bbamcr.2013.12.011 PMC4317292 24370777

[114] Ellis TN Kuehn MJ. Virulence and immunomodulatory roles of bacterial outer membrane vesicles. Microbiol Mol Biol Rev (2010) 74(1):81–94. doi: 10.1128/MMBR.00031-09 20197500 PMC2832350

[115] Zhang Z Liu D Liu S Zhang S Pan Y. The role of porphyromonas gingivalis outer membrane vesicles in periodontal disease and related systemic diseases. Front Cell Infect Microbiol (2020) 10:585917. doi: 10.3389/fcimb.2020.585917 33585266 PMC7877337

[116] Briaud P Carroll RK. Extracellular vesicle biogenesis and functions in gram-positive bacteria. Infect Immun (2020) 88(12):e00433–20. doi: 10.1128/IAI.00433-20 PMC7671900 32989035

[117] Kolaczkowska E Kubes P. Neutrophil recruitment and function in health and inflammation. Nat Rev Immunol (2013) 13(3):159–75. doi: 10.1038/nri3399 23435331

[118] Amano A. Disruption of epithelial barrier and impairment of cellular function by porphyromonas gingivalis. Front Biosci (2007) 12:3965–74. doi: 10.2741/2363 17485350

[119] Ho MH Chen CH Goodwin JS Wang BY Xie H. Functional advantages of porphyromonas gingivalis vesicles. PloS One (2015) 10(4):e0123448. doi: 10.1371/journal.pone.0123448 25897780 PMC4405273

[120] Furuta N Takeuchi H Amano A. Entry of porphyromonas gingivalis outer membrane vesicles into epithelial cells causes cellular functional impairment. Infect Immun (2009) 77(11):4761–70. doi: 10.1128/IAI.00841-09 PMC2772519 19737899

[121] Furuta N Tsuda K Omori H Yoshimori T Yoshimura F Amano A. Porphyromonas gingivalis outer membrane vesicles enter human epithelial cells *via* an endocytic pathway and are sorted to lysosomal compartments. Infect Immun (2009) 77(10):4187–96. doi: 10.1128/IAI.00009-09 PMC2747946 19651865

[122] Chi B Qi M Kuramitsu HK. Role of dentilisin in treponema denticola epithelial cell layer penetration. Res Microbiol (2003) 154(9):637–43. doi: 10.1016/j.resmic.2003.08.001 14596901

[123] Bartruff JB Yukna RA Layman DL. Outer membrane vesicles from porphyromonas gingivalis affect the growth and function of cultured human gingival fibroblasts and umbilical vein endothelial cells. J Periodontol (2005) 76(6):972–9. doi: 10.1902/jop.2005.76.6.972 15948693

[124] Kamaguchi A Nakayama K Ichiyama S Nakamura R Watanabe T Ohta M. Effect of porphyromonas gingivalis vesicles on coaggregation of staphylococcus aureus to oral microorganisms. Curr Microbiol (2003) 47(6):485–91. doi: 10.1007/s00284-003-4069-6 14756532

[125] Grenier D. Porphyromonas gingivalis outer membrane vesicles mediate coaggregation and piggybacking of treponema denticola and lachnoanaerobaculum saburreum. Int J Dent. (2013) 2013:305476. doi: 10.1155/2013/305476 23365576 PMC3556864

[126] Orth RK O'Brien-Simpson NM Dashper SG Reynolds EC. Synergistic virulence of porphyromonas gingivalis and treponema denticola in a murine periodontitis model. Mol Oral Microbiol (2011) 26(4):229–40. doi: 10.1111/j.2041-1014.2011.00612.x 21729244

[127] Inagaki S Onishi S Kuramitsu HK Sharma A. Porphyromonas gingivalis vesicles enhance attachment, and the leucine-rich repeat BspA protein is required for invasion of epithelial cells by "Tannerella forsythia". Infect Immun (2006) 74(9):5023–8. doi: 10.1128/IAI.00062-06 PMC1594857 16926393

[128] Veith PD Chen YY Gorasia DG Chen D Glew MD O'Brien-Simpson NM. Porphyromonas gingivalis outer membrane vesicles exclusively contain outer membrane and periplasmic proteins and carry a cargo enriched with virulence factors. J Proteome Res (2014) 13(5):2420–32. doi: 10.1021/pr401227e 24620993

[129] Cecil JD Sirisaengtaksin N O'Brien-Simpson NM Krachler AM. Outer membrane vesicle-host cell interactions. Microbiol Spectr (2019) 7(1):10.1128. doi: 10.1128/microbiolspec.PSIB-0001-2018. PMC6352913 30681067

[130] Choi JW Kim SC Hong SH Lee HJ. Secretable small RNAs *via* outer membrane vesicles in periodontal pathogens. J Dent Res (2017) 96(4):458–66. doi: 10.1177/0022034516685071 28068479

[131] Waller T Kesper L Hirschfeld J Dommisch H Kolpin J Oldenburg J. Porphyromonas gingivalis outer membrane vesicles induce selective tumor necrosis factor tolerance in a toll-like receptor 4- and mTOR-dependent manner. Infect Immun (2016) 84(4):1194–204. doi: 10.1128/IAI.01390-15 PMC4807478 26857578

[132] du Teil Espina M Fu Y van der Horst D Hirschfeld C Lopez-Alvarez M Mulder LM. Coating and corruption of human neutrophils by bacterial outer membrane vesicles. Microbiol Spectr. (2022) 10(5):e0075322. doi: 10.1128/spectrum.00753-22 36000865 PMC9602476

[133] Pelletier M Maggi L Micheletti A Lazzeri E Tamassia N Costantini C. Evidence for a cross-talk between human neutrophils and Th17 cells. Blood. (2010) 115(2):335–43. doi: 10.1182/blood-2009-04-216085 19890092

[134] Noguchi K Ishikawa I. The roles of cyclooxygenase-2 and prostaglandin E2 in periodontal disease. Periodontol 2000 (2007) 43(1):43:85–101. doi: 10.1111/j.1600-0757.2006.00170.x 17214837

[135] Kou Y Inaba H Kato T Tagashira M Honma D Kanda T. Inflammatory responses of gingival epithelial cells stimulated with porphyromonas gingivalis vesicles are inhibited by hop-associated polyphenols. J Periodontol (2008) 79(1):174–80. doi: 10.1902/jop.2008.070364 18166108

[136] Uemura Y Hiroshima Y Tada A Murakami K Yoshida K Inagaki Y. Porphyromonas gingivalis outer membrane vesicles stimulate gingival epithelial cells to induce pro-inflammatory cytokines via the MAPK and STING pathways. Biomedicines (2022) 10(10):2643. doi: 10.3390/biomedicines10102643 36289904 PMC9599832

[137] Fleetwood AJ Lee MKS Singleton W Achuthan A Lee MC O'Brien-Simpson NM. Metabolic remodeling, inflammasome activation, and pyroptosis in macrophages stimulated by porphyromonas gingivalis and its outer membrane vesicles. Front Cell Infect Microbiol (2017) 7:351. doi: 10.3389/fcimb.2017.00351 28824884 PMC5543041

[138] Lim Y Kim HY An SJ Choi BK. Activation of bone marrow-derived dendritic cells and CD4(+) T cell differentiation by outer membrane vesicles of periodontal pathogens. J Oral Microbiol (2022) 14(1):2123550. doi: 10.1080/20002297.2022.2123550 36312320 PMC9616074

[139] Friedrich V Gruber C Nimeth I Pabinger S Sekot G Posch G. Outer membrane vesicles of tannerella forsythia: biogenesis, composition, and virulence. Mol Oral Microbiol (2015) 30(6):451–73. doi: 10.1111/omi.12104 PMC4604654 25953484

[140] Dominy SS Lynch C Ermini F Benedyk M Marczyk A Konradi A. Porphyromonas gingivalis in alzheimer's disease brains: Evidence for disease causation and treatment with small-molecule inhibitors. Sci Adv (2019) 5(1):eaau3333. doi: 10.1126/sciadv.aau3333. 30746447 PMC6357742

[141] Gonzalez-Sanmiguel J Schuh C Munoz-Montesino C Contreras-Kallens P Aguayo LG Aguayo S. Complex interaction between resident microbiota and misfolded proteins: Role in neuroinflammation and neurodegeneration. Cells (2020) 9(11). doi: 10.3390/cells9112476. PMC7697492 33203002

[142] Farrugia C Stafford GP Murdoch C. Porphyromonas gingivalis outer membrane vesicles increase vascular permeability. J Dent Res (2020) 99(13):1494–501. doi: 10.1177/0022034520943187 PMC7684789 32726180

[143] Dean SN Leary DH Sullivan CJ Oh E Walper SA. Isolation and characterization of lactobacillus-derived membrane vesicles. Sci Rep (2019) 9(1):877. doi: 10.1038/s41598-018-37120-6 30696852 PMC6351534

[144] Diaz-Garrido N Badia J Baldoma L. Microbiota-derived extracellular vesicles in interkingdom communication in the gut. J Extracell Vesicles. (2021) 10(13):e12161. doi: 10.1002/jev2.12161 34738337 PMC8568775

[145] Chen Q Huang G Wu W Wang J Hu J Mao J. A hybrid eukaryotic-prokaryotic nanoplatform with photothermal modality for enhanced antitumor vaccination. Adv Mater (2020) 32(16):e1908185. doi: 10.1002/adma.201908185 32108390

[146] Veith PD Glew MD Gorasia DG Chen D O'Brien-Simpson NM Reynolds EC. Localization of outer membrane proteins in treponema denticola by quantitative proteome analyses of outer membrane vesicles and cellular fractions. J Proteome Res (2019) 18(4):1567–81. doi: 10.1021/acs.jproteome.8b00860 30761904

[147] Nakao R Hasegawa H Dongying B Ohnishi M Senpuku H. Assessment of outer membrane vesicles of periodontopathic bacterium porphyromonas gingivalis as possible mucosal immunogen. Vaccine. (2016) 34(38):4626–34. doi: 10.1016/j.vaccine.2016.06.016 27461458

[148] Nakao R Hasegawa H Ochiai K Takashiba S Ainai A Ohnishi M. Outer membrane vesicles of porphyromonas gingivalis elicit a mucosal immune response. PloS One (2011) 6(10):e26163. doi: 10.1371/journal.pone.0026163 22022548 PMC3193504

[149] Mehanny M Lehr CM Fuhrmann G. Extracellular vesicles as antigen carriers for novel vaccination avenues. Adv Drug Delivery Rev (2021) 173:164–80. doi: 10.1016/j.addr.2021.03.016 33775707

[150] Elsayed R Elashiry M Liu Y El-Awady A Hamrick M Cutler CW. Porphyromonas gingivalis provokes exosome secretion and paracrine immune senescence in bystander dendritic cells. Front Cell Infect Microbiol (2021) 11:669989. doi: 10.3389/fcimb.2021.669989 34141629 PMC8204290

[151] Elsayed R Elashiry M Liu Y Morandini AC El-Awady A Elashiry MM. Microbially-induced exosomes from dendritic cells promote paracrine immune senescence: Novel mechanism of bone degenerative disease in mice. Aging Dis (2023) 14(1):136–51. doi: 10.14336/AD.2022.0623 PMC9937696 36818565

[152] Bi J Koivisto L Owen G Huang P Wang Z Shen Y. Epithelial microvesicles promote an inflammatory phenotype in fibroblasts. J Dent Res (2016) 95(6):680–8. doi: 10.1177/0022034516633172 26912223

[153] Zhao M Dai W Wang H Xue C Feng J He Y. Periodontal ligament fibroblasts regulate osteoblasts by exosome secretion induced by inflammatory stimuli. Arch Oral Biol (2019) 105:27–34. doi: 10.1016/j.archoralbio.2019.06.002 31247478

[154] Dawes C Wong DTW. Role of saliva and salivary diagnostics in the advancement of oral health. J Dent Res (2019) 98(2):133–41. doi: 10.1177/0022034518816961 PMC6900436 30782091

[155] Helmerhorst EJ Oppenheim FG. Saliva: a dynamic proteome. J Dent Res (2007) 86(8):680–93. doi: 10.1177/154405910708600802 17652194

[156] Novakovic N Todorovic T Rakic M Milinkovic I Dozic I Jankovic S. Salivary antioxidants as periodontal biomarkers in evaluation of tissue status and treatment outcome. J Periodontal Res (2014) 49(1):129–36. doi: 10.1111/jre.12088 23710550

[157] Chaparro Padilla A Weber Aracena L Realini Fuentes O Albers Busquetts D Hernandez Rios M Ramirez Lobos V. Molecular signatures of extracellular vesicles in oral fluids of periodontitis patients. Oral Dis (2020) 26(6):1318–25. doi: 10.1111/odi.13338 32232928

[158] Sanders AE Slade GD Fitzsimmons TR Bartold PM. Physical activity, inflammatory biomarkers in gingival crevicular fluid and periodontitis. J Clin Periodontol (2009) 36(5):388–95. doi: 10.1111/j.1600-051X.2009.01394.x 19419437

[159] Baima G Corana M Iaderosa G Romano F Citterio F Meoni G. Metabolomics of gingival crevicular fluid to identify biomarkers for periodontitis: A systematic review with meta-analysis. J Periodontal Res (2021) 56(4):633–45. doi: 10.1111/jre.12872 33710624

[160] Huang X Hu X Zhao M Zhang Q. Analysis of salivary exosomal proteins in young adults with severe periodontitis. Oral Dis (2020) 26(1):173–81. doi: 10.1111/odi.13217 31630466

[161] Han P Bartold PM Salomon C Ivanovski S. Salivary outer membrane vesicles and DNA methylation of small extracellular vesicles as biomarkers for periodontal status: A pilot study. Int J Mol Sci (2021) 22(5):2423. doi: 10.3390/ijms22052423 33670900 PMC7957785

[162] Tobon-Arroyave SI Celis-Mejia N Cordoba-Hidalgo MP Isaza-Guzman DM. Decreased salivary concentration of CD9 and CD81 exosome-related tetraspanins may be associated with the periodontal clinical status. J Clin Periodontol (2019) 46(4):470–80. doi: 10.1111/jcpe.13099 30825338

[163] Nik Mohamed Kamal NNS Awang RAR Mohamad S Shahidan WNS. Plasma- and saliva exosome profile reveals a distinct MicroRNA signature in chronic periodontitis. Front Physiol (2020) 11:587381. doi: 10.3389/fphys.2020.587381 33329037 PMC7733931

[164] Xia Y Zhou K Sun M Shu R Qian J Xie Y. The miR-223-3p regulates pyroptosis through NLRP3-caspase 1-GSDMD signal axis in periodontitis. Inflammation. (2021) 44(6):2531–42. doi: 10.1007/s10753-021-01522-y 34637033

[165] Yu J Lin Y Xiong X Li K Yao Z Dong H. Detection of exosomal PD-L1 RNA in saliva of patients with periodontitis. Front Genet (2019) 10:202. doi: 10.3389/fgene.2019.00202 30923536 PMC6426748

[166] Han P Bartold PM Salomon C Ivanovski S. Salivary small extracellular vesicles associated miRNAs in periodontal status-a pilot study. Int J Mol Sci (2020) 21(8):2809. doi: 10.3390/ijms21082809 32316600 PMC7215885

[167] O'Brien-Simpson NM Burgess K Brammar GC Darby IB Reynolds EC. Development and evaluation of a saliva-based chair-side diagnostic for the detection of porphyromonas gingivalis. J Oral Microbiol (2015) 7:29129. doi: 10.3402/jom.v7.29129 26387647 PMC4576421

[168] Fatima T Khurshid Z Rehman A Imran E Srivastava KC Shrivastava D. Gingival crevicular fluid (GCF): A diagnostic tool for the detection of periodontal health and diseases. Molecules (2021) 26(5):1208. doi: 10.3390/molecules26051208. 33668185 PMC7956529

[169] Fitzsimmons TR Sanders AE Bartold PM Slade GD. Local and systemic biomarkers in gingival crevicular fluid increase odds of periodontitis. J Clin Periodontol (2010) 37(1):30–6. doi: 10.1111/j.1600-051X.2009.01506.x 19995404

[170] Gomar-Vercher S Simon-Soro A Montiel-Company JM Almerich-Silla JM Mira A. Stimulated and unstimulated saliva samples have significantly different bacterial profiles. PloS One (2018) 13(6):e0198021. doi: 10.1371/journal.pone.0198021 29856779 PMC5983451

[171] Han P Ivanovski S. Effect of saliva collection methods on the detection of periodontium-related genetic and epigenetic biomarkers-a pilot study. Int J Mol Sci (2019) 20(19):4729. doi: 10.3390/ijms20194729 31554202 PMC6801527

[172] Engebretson SP Grbic JT Singer R Lamster IB. GCF IL-1beta profiles in periodontal disease. J Clin Periodontol (2002) 29(1):48–53. doi: 10.1034/j.1600-051x.2002.290108.x 11846849

[173] Zlotogorski-Hurvitz A Dayan D Chaushu G Korvala J Salo T Sormunen R. Human saliva-derived exosomes: comparing methods of isolation. J Histochem Cytochem (2015) 63(3):181–9. doi: 10.1369/0022155414564219 PMC4340734 25473095

[174] Buduneli N Kinane DF. Host-derived diagnostic markers related to soft tissue destruction and bone degradation in periodontitis. J Clin Periodontol (2011) 38 Suppl 11:85–105. doi: 10.1111/j.1600-051X.2010.01670.x 21323706

[175] Sun X Gao J Meng X Lu X Zhang L Chen R. Polarized macrophages in periodontitis: Characteristics, function, and molecular signaling. Front Immunol (2021) 12:763334. doi: 10.3389/fimmu.2021.763334 34950140 PMC8688840

[176] Sima C Glogauer M. Macrophage subsets and osteoimmunology: tuning of the immunological recognition and effector systems that maintain alveolar bone. Periodontol 2000. (2013) 63(1):80–101. doi: 10.1111/prd.12032 23931056

[177] Yang J Zhu Y Duan D Wang P Xin Y Bai L. Enhanced activity of macrophage M1/M2 phenotypes in periodontitis. Arch Oral Biol (2018) 96:234–42. doi: 10.1016/j.archoralbio.2017.03.006 28351517

[178] Zhang Q Chen B Yan F Guo J Zhu X Ma S. Interleukin-10 inhibits bone resorption: a potential therapeutic strategy in periodontitis and other bone loss diseases. BioMed Res Int (2014) 2014:284836. doi: 10.1155/2014/284836 24696846 PMC3947664

[179] Garaicoa-Pazmino C Fretwurst T Squarize CH Berglundh T Giannobile WV Larsson L. Characterization of macrophage polarization in periodontal disease. J Clin Periodontol (2019) 46(8):830–9. doi: 10.1111/jcpe.13156 31152604

[180] Kang M Huang CC Gajendrareddy P Lu Y Shirazi S Ravindran S. Extracellular vesicles from TNFalpha preconditioned MSCs: Effects on immunomodulation and bone regeneration. Front Immunol (2022) 13:878194. doi: 10.3389/fimmu.2022.878194 35585987 PMC9108364

[181] Zhang Y Chen J Fu H Kuang S He F Zhang M. Exosomes derived from 3D-cultured MSCs improve therapeutic effects in periodontitis and experimental colitis and restore the Th17 cell/Treg balance in inflamed periodontium. Int J Oral Sci (2021) 13(1):43. doi: 10.1038/s41368-021-00150-4 34907166 PMC8671433

[182] Xu R Zhang F Chai R Zhou W Hu M Liu B. Exosomes derived from pro-inflammatory bone marrow-derived mesenchymal stem cells reduce inflammation and myocardial injury *via* mediating macrophage polarization. J Cell Mol Med (2019) 23(11):7617–31. doi: 10.1111/jcmm.14635 PMC6815833 31557396

[183] Ye Q Xu H Liu S Li Z Zhou J Ding F. Apoptotic extracellular vesicles alleviate pg-LPS induced inflammatory responses of macrophages *via* AMPK/SIRT1/NF-kappaB pathway and inhibit osteoclast formation. J Periodontol (2022) 93(11):1738–51. doi: 10.1002/JPER.21-0657 35499816

[184] Yue C Cao J Wong A Kim JH Alam S Luong G. Human bone marrow stromal cell exosomes ameliorate periodontitis. J Dent Res (2022) 101(9):1110–8. doi: 10.1177/00220345221084975 PMC9305845 35356822

[185] Nakao Y Fukuda T Zhang Q Sanui T Shinjo T Kou X. Exosomes from TNF-alpha-treated human gingiva-derived MSCs enhance M2 macrophage polarization and inhibit periodontal bone loss. Acta Biomater. (2021) 122:306–24. doi: 10.1016/j.actbio.2020.12.046 PMC7897289 33359765

[186] Wang R Ji Q Meng C Liu H Fan C Lipkind S. Role of gingival mesenchymal stem cell exosomes in macrophage polarization under inflammatory conditions. Int Immunopharmacol. (2020) 81:106030. doi: 10.1016/j.intimp.2019.106030 31796385

[187] Zarubova J Hasani-Sadrabadi MM Dashtimoghadam E Zhang X Ansari S Li S. Engineered delivery of dental stem-Cell-Derived extracellular vesicles for periodontal tissue regeneration. Adv Healthc Mater (2022) 11(12):e2102593. doi: 10.1002/adhm.202102593 35191610 PMC9233004

[188] Zheng Y Dong C Yang J Jin Y Zheng W Zhou Q. Exosomal microRNA-155-5p from PDLSCs regulated Th17/Treg balance by targeting sirtuin-1 in chronic periodontitis. J Cell Physiol (2019) 234(11):20662–74. doi: 10.1002/jcp.28671 31016751

[189] Kang H Lee MJ Park SJ Lee MS. Lipopolysaccharide-preconditioned periodontal ligament stem cells induce M1 polarization of macrophages through extracellular vesicles. Int J Mol Sci (2018) 19(12):3843. doi: 10.3390/ijms19123843. 30513870 PMC6321485

[190] Elashiry M Elsayed R Cutler CW. Exogenous and endogenous dendritic cell-derived exosomes: Lessons learned for immunotherapy and disease pathogenesis. Cells (2021) 11(1):115. doi: 10.3390/cells11010115 35011677 PMC8750541

[191] El-Awady AR Elashiry M Morandini AC Meghil MM Cutler CW. Dendritic cells a critical link to alveolar bone loss and systemic disease risk in periodontitis: Immunotherapeutic implications. Periodontol 2000. (2022) 89(1):41–50. doi: 10.1111/prd.12428 35244951 PMC9018591

[192] Elashiry M Elashiry MM Elsayed R Rajendran M Auersvald C Zeitoun R. Dendritic cell derived exosomes loaded with immunoregulatory cargo reprogram local immune responses and inhibit degenerative bone disease in vivo. J Extracell Vesicles (2020) 9(1):1795362. doi: 10.1080/20013078.2020.1795362 32944183 PMC7480413

[193] Ramseier CA Rasperini G Batia S Giannobile WV. Advanced reconstructive technologies for periodontal tissue repair. Periodontol 2000. (2012) 59(1):185–202. doi: 10.1111/j.1600-0757.2011.00432.x 22507066 PMC3335769

[194] Zhu B Liu W Zhang H Zhao X Duan Y Li D. Tissue-specific composite cell aggregates drive periodontium tissue regeneration by reconstructing a regenerative microenvironment. J Tissue Eng Regener Med (2017) 11(6):1792–805. doi: 10.1002/term.2077 26455905

[195] Wei F Li Z Crawford R Xiao Y Zhou Y. Immunoregulatory role of exosomes derived from differentiating mesenchymal stromal cells on inflammation and osteogenesis. J Tissue Eng Regener Med (2019) 13(11):1978–91. doi: 10.1002/term.2947 31359542

[196] Lan Y Xie H Jin Q Zhao X Shi Y Zhou Y. Extracellular vesicles derived from neural EGFL-like 1-modified mesenchymal stem cells improve acellular bone regeneration *via* the miR-25-5p-SMAD2 signaling axis. Bioact Mater (2022) 17:457–70. doi: 10.1016/j.bioactmat.2022.01.019 PMC8961279 35386450

[197] Huang CC Kang M Lu Y Shirazi S Diaz JI Cooper LF. Functionally engineered extracellular vesicles improve bone regeneration. Acta Biomater. (2020) 109:182–94. doi: 10.1016/j.actbio.2020.04.017 PMC8040700 32305445

[198] Li M Xing X Huang H Liang C Gao X Tang Q. BMSC-derived ApoEVs promote craniofacial bone repair *via* ROS/JNK signaling. J Dent Res (2022) 101(6):714–23. doi: 10.1177/00220345211068338 35114838

[199] Qin Y Wang L Gao Z Chen G Zhang C. Bone marrow stromal/stem cell-derived extracellular vesicles regulate osteoblast activity and differentiation in vitro and promote bone regeneration *in vivo* . Sci Rep (2016) 6:21961. doi: 10.1038/srep21961 26911789 PMC4766421

[200] Gonzalez-King H Garcia NA Ontoria-Oviedo I Ciria M Montero JA Sepulveda P. Hypoxia inducible factor-1alpha potentiates jagged 1-mediated angiogenesis by mesenchymal stem cell-derived exosomes. Stem Cells (2017) 35(7):1747–59. doi: 10.1002/stem.2618 28376567

[201] Cheng P Cao T Zhao X Lu W Miao S Ning F. Nidogen1-enriched extracellular vesicles accelerate angiogenesis and bone regeneration by targeting myosin-10 to regulate endothelial cell adhesion. Bioact Mater (2022) 12:185–97. doi: 10.1016/j.bioactmat.2021.10.021 PMC8897190 35310379

[202] Xie H Wang Z Zhang L Lei Q Zhao A Wang H. Extracellular vesicle-functionalized decalcified bone matrix scaffolds with enhanced pro-angiogenic and pro-bone regeneration activities. Sci Rep (2017) 7:45622. doi: 10.1038/srep45622 28367979 PMC5377422

[203] Mohammed E Khalil E Sabry D. Effect of adipose-derived stem cells and their exo as adjunctive therapy to nonsurgical periodontal treatment: A histologic and histomorphometric study in rats. Biomolecules (2018) 10:8(4). doi: 10.3390/biom8040167 PMC6316309 30544734

[204] Yang Y Zhang B Yang Y Peng B Ye R. PLGA containing human adipose-derived stem cell-derived extracellular vesicles accelerates the repair of alveolar bone defects *via* transfer of CGRP. Oxid Med Cell Longev (2022) 2022:4815284. doi: 10.1155/2022/4815284 35726333 PMC9206573

[205] Li W Liu Y Zhang P Tang Y Zhou M Jiang W. Tissue-engineered bone immobilized with human adipose stem cells-derived exosomes promotes bone regeneration. ACS Appl Mater Interfaces. (2018) 10(6):5240–54. doi: 10.1021/acsami.7b17620 29359912

[206] Yan C Li N Xiao T Ye X Fu L Ye Y. Extracellular vesicles from the inflammatory microenvironment regulate the osteogenic and odontogenic differentiation of periodontal ligament stem cells by miR-758-5p/LMBR1/BMP2/4 axis. J Transl Med (2022) 20(1):208. doi: 10.1186/s12967-022-03412-9 35562763 PMC9103284

[207] Shimizu Y Takeda-Kawaguchi T Kuroda I Hotta Y Kawasaki H Hariyama T. Exosomes from dental pulp cells attenuate bone loss in mouse experimental periodontitis. J Periodontal Res (2022) 57(1):162–72. doi: 10.1111/jre.12949 34826339

[208] Jin Q Li P Yuan K Zhao F Zhu X Zhang P. Extracellular vesicles derived from human dental pulp stem cells promote osteogenesis of adipose-derived stem cells *via* the MAPK pathway. J Tissue Eng. (2020) 11:2041731420975569. doi: 10.1177/2041731420975569 33312494 PMC7716067

[209] Xian X Gong Q Li C Guo B Jiang H. Exosomes with highly angiogenic potential for possible use in pulp regeneration. J Endod. (2018) 44(5):751–8. doi: 10.1016/j.joen.2017.12.024 29426641

[210] Zhou H Li X Yin Y He XT An Y Tian BM. The proangiogenic effects of extracellular vesicles secreted by dental pulp stem cells derived from periodontally compromised teeth. Stem Cell Res Ther (2020) 11(1):110. doi: 10.1186/s13287-020-01614-w 32143712 PMC7060605

[211] Imanishi Y Hata M Matsukawa R Aoyagi A Omi M Mizutani M. Efficacy of extracellular vesicles from dental pulp stem cells for bone regeneration in rat calvarial bone defects. Inflammation Regen. (2021) 41(1):12. doi: 10.1186/s41232-021-00163-w PMC8048358 33853679

[212] Wang M Li J Ye Y He S Song J. SHED-derived conditioned exosomes enhance the osteogenic differentiation of PDLSCs *via* wnt and BMP signaling in vitro. Differentiation (2020) 111:1–11. doi: 10.1016/j.diff.2019.10.003. 31630077

[213] Wei J Song Y Du Z Yu F Zhang Y Jiang N. Exosomes derived from human exfoliated deciduous teeth ameliorate adult bone loss in mice through promoting osteogenesis. J Mol Histol. (2020) 51(4):455–66. doi: 10.1007/s10735-020-09896-3 32656578

[214] Pizzicannella J Gugliandolo A Orsini T Fontana A Ventrella A Mazzon E. Engineered extracellular vesicles from human periodontal-ligament stem cells increase VEGF/VEGFR2 expression during bone regeneration. Front Physiol (2019) 10:512. doi: 10.3389/fphys.2019.00512 31114512 PMC6503111

[215] Diomede F D'Aurora M Gugliandolo A Merciaro I Ettorre V Bramanti A. A novel role in skeletal segment regeneration of extracellular vesicles released from periodontal-ligament stem cells. Int J Nanomedicine. (2018) 13:3805–25. doi: 10.2147/IJN.S162836 PMC6029600 29988728

[216] Zhao B Chen Q Zhao L Mao J Huang W Han X. Periodontal ligament stem cell-derived small extracellular vesicles embedded in matrigel enhance bone repair through the adenosine receptor signaling pathway. Int J Nanomedicine. (2022) 17:519–36. doi: 10.2147/IJN.S346755 PMC8819539 35140462

[217] Jiang S Xu L. Exosomes from gingival mesenchymal stem cells enhance migration and osteogenic differentiation of pre-osteoblasts. Pharmazie. (2020) 75(11):576–80. doi: 10.1691/ph.2020.0652 33239132

[218] Diomede F Gugliandolo A Cardelli P Merciaro I Ettorre V Traini T. Three-dimensional printed PLA scaffold and human gingival stem cell-derived extracellular vesicles: a new tool for bone defect repair. Stem Cell Res Ther (2018) 9(1):104. doi: 10.1186/s13287-018-0850-0 29653587 PMC5899396

[219] Ma L Rao N Jiang H Dai Y Yang S Yang H. Small extracellular vesicles from dental follicle stem cells provide biochemical cues for periodontal tissue regeneration. Stem Cell Res Ther (2022) 13(1):92. doi: 10.1186/s13287-022-02767-6 35241181 PMC8895915

[220] Liu Z Wang S Huo N Yang S Shi Q Xu J. Extracellular vesicles: A potential future strategy for dental and maxillofacial tissue repair and regeneration. Front Physiol (2022) 13:1012241. doi: 10.3389/fphys.2022.1012241 36479350 PMC9719951

[221] Tsiapalis D O'Driscoll L. Mesenchymal stem cell derived extracellular vesicles for tissue engineering and regenerative medicine applications. Cells (2020) 9(4):991. doi: 10.3390/cells9040991 32316248 PMC7226943

[222] Novello S Pellen-Mussi P Jeanne S. Mesenchymal stem cell-derived small extracellular vesicles as cell-free therapy: Perspectives in periodontal regeneration. J Periodontal Res (2021) 56(3):433–42. doi: 10.1111/jre.12866 33641196

[223] Lener T Gimona M Aigner L Borger V Buzas E Camussi G. Applying extracellular vesicles based therapeutics in clinical trials - an ISEV position paper. J Extracell Vesicles. (2015) 4:30087. doi: 10.3402/jev.v4.30087 26725829 PMC4698466

[224] Ng CY Kee LT Al-Masawa ME Lee QH Subramaniam T Kok D. Scalable production of extracellular vesicles and its therapeutic values: A review. Int J Mol Sci (2022) 23(14). doi: 10.3390/ijms23147986 PMC9315612 35887332

